# Commensal Bacteria in the Cystic Fibrosis Airway Microbiome Reduce *P. aeruginosa* Induced Inflammation

**DOI:** 10.3389/fcimb.2022.824101

**Published:** 2022-01-31

**Authors:** Andrew Tony-Odigie, Leonie Wilke, Sébastien Boutin, Alexander H. Dalpke, Buqing Yi

**Affiliations:** ^1^ Institute of Medical Microbiology and Virology, University Hospital Carl Gustav Carus, Technische Universität Dresden, Dresden, Germany; ^2^ Translational Lung Research Center (TLRC), Member of the German Center for Lung Research (DZL), Heidelberg, Germany; ^3^ Department of Infectious Diseases, Medical Microbiology and Hygiene, University of Heidelberg, Heidelberg, Germany

**Keywords:** *Pseudomonas aeruginosa*, beneficial commensal, inflammation inhibition, airway microbiome, cystic fibrosis

## Abstract

Chronic *Pseudomonas aeruginosa* infections play an important role in the progress of lung disease in patients suffering from cystic fibrosis (CF). Recent studies indicate that polymicrobial microbiome profiles in the airway are associated with less inflammation. Thus, the hypothesis was raised that certain commensal bacteria might protect the host from inflammation. We therefore performed a screening study with commensals isolated from CF airway microbiome samples to identify potential beneficial commensals. We isolated more than 80 aerobic or facultative anaerobic commensal strains, including strains from genera *Streptococcus*, *Neisseria*, *Actinomyces*, *Corynebacterium*, *Dermabacter*, *Micrococcus* and *Rothia*. Through a screening experiment of co-infection in human epithelial cell lines, we identified multiple commensal strains, especially strains belonging to *Streptococcus mitis*, that reduced *P. aeruginosa* triggered inflammatory responses. The results were confirmed by co-infection experiments in *ex-vivo* precision cut lung slices (PCLS) from mice. The underlying mechanisms of the complex host-pathogen-commensal crosstalk were investigated from both the host and the bacterial sides with a focus on *S. mitis*. Transcriptome changes in the host in response to co-infection and mono-infection were evaluated, and the results indicated that several signalling pathways mediating inflammatory responses were downregulated by co-infection with *S. mitis and P. aeruginosa* compared to *P. aeruginosa* mono-infection, such as neutrophil extracellular trap formation. The genomic differences among *S. mitis* strains with and without protective effects were investigated by whole genome sequencing, revealing genes only present in the *S. mitis* strains showing protective effects. In summary, through both *in vitro* and *ex vivo* studies, we could identify a variety of commensal strains that may reduce host inflammatory responses induced by *P. aeruginosa* infection. These findings support the hypothesis that CF airway commensals may protect the host from inflammation.

## Introduction

Cystic fibrosis (CF) is caused by CFTR (cystic fibrosis transmembrane regulator) mutations (Vankeerberghen et al., 2002; Goss and Burns, 2007), which lead to dysfunction in the chloride channel and induce mucus hypersecretion and impaired mucociliary clearance. These conditions favor microbial colonization and may trigger increased stimulation of the immune system, resulting in long-lasting hyperactive inflammatory responses (Kreda et al., 2012; Mall and Hartl, 2014; Elborn, 2016). Chronic infection and inflammation contribute to a decline in lung function and eventually result in lung failure (Gellatly and Hancock, 2013). Thus, infection, inflammation and tissue damage comprise a cycle causing progressive loss of lung function, and infection plays a critical role in initiating this cycle. Investigating the airway microbial community structure in people with CF is therefore important to understand at which steps this cycle can be disrupted.

It has been revealed by several studies that the airway microbiome can be either polymicrobial, often showing prevalence of oral commensal bacteria, or can be dominated by one typical CF pathogen, such as *Pseudomonas aeruginosa* or other lung pathogenic bacterial species. Importantly, samples dominated by one typical CF pathogen were associated with diminished microbial diversity and positively correlated to reductions in the lung function and increase in inflammation (van der Gast et al., 2011; Brown et al., 2014; Boutin et al., 2015; Coburn et al., 2015; Flight et al., 2015; Zemanick et al., 2017). In contrast, increased abundance of commensal bacteria in the airway microbiome was associated with more preserved lung functions and reduced inflammation (Boutin et al., 2017; Frey et al., 2021). Yet, it is still unknown how to prevent the decrease of bacterial diversity in people with CF at an early stage. The complicated interactions between pathogenic and commensal bacteria in the CF microbiome community are still underexplored, and different kinds of interactions, synergistic and antagonistic ones, have been reported (Whiley et al., 2015; Scott and O'Toole, 2019).

The results of multiple previous CF microbiome studies raised the hypothesis that certain commensal bacteria might interfere with pathogenic bacteria and protect the host from inflammation (Filkins et al., 2012; Coburn et al., 2015; Flight et al., 2015; Boutin et al., 2017; Muhlebach et al., 2018; Ronan et al., 2018; Frey et al., 2021). In particular, it has been shown that the strongest predictor of CF patient stability was a high fraction of Streptococci in the patient sputum samples (Filkins et al., 2012). We therefore performed a screening study with commensal bacterial strains isolated from airway samples of people suffering from CF to identify airway commensal bacteria that are able to reduce pro-inflammatory responses triggered by *P. aeruginosa* infection.

## Materials and Methods

### Isolation of Aerobic or Facultative Anaerobic Commensal Microbes

Sputum samples from people with cystic fibrosis were streaked on Columbia Blood Agar (CBA) (Thermo Fisher Scientific, Waltham, USA) and cultured aerobically for 24h in order to isolate aerobic or facultative anaerobic strains. Isolated bacteria were identified with MALDI Biotyper System (Bruker Daltonik GmbH & Co. KG, Bremen, Germany) based on Matrix-Assisted Laser Desorption/Ionization Time-Of-Flight (MALDI-TOF) mass spectrometry. Strains belonging to species that are not typically considered as pathogens and recognized as part of the commensal airway microbiota according to literature information [e.g. (Man et al., 2017; de Steenhuijsen Piters et al., 2020)] were stored in skimmed milk at -20°C and used for further analysis (approved by the ethical board of the University Hospital Heidelberg with the ethical vote: S-370/2011). The commensal bacteria that were isolated in this study are from genera *Streptococcus* (Man et al., 2017; de Steenhuijsen Piters et al., 2020), *Rothia* (Man et al., 2017; de Steenhuijsen Piters et al., 2020), *Corynebacterium* (Man et al., 2017), *Neisseria* (de Steenhuijsen Piters et al., 2020), *Actinomyces* (Hilty et al., 2010; Kramer et al., 2015), *Dermabacter* (Leal et al., 2016) and *Micrococcus* (Zakharkina et al., 2013). The exact assembly of the collection is described in the *Results* section.

### Cell Culture

Human bronchial epithelial BEAS-2B cells (ref: CRL-9609™, ATCC^®^, Manassas, VA, USA) were cultured in RPMI growth medium, supplemented with 10% FCS and 1% penicillin/streptomycin at 37°C, 5% CO2 in a humidified incubator.

### Infection of Cells

For infection of BEAS-2B cells, 50,000 BEAS-2B cells were seeded in 96-well-plates in RPMI growth medium, supplemented with 10% FCS. Twenty-four hours later, BEAS-2B were treated with either mono-infection or co-infection. For mono-infection, *Pseudomonas aeruginosa* strain PAO1 (DSMZ22644) or one commensal at a multiplicity of infection of 10 (MOI 10) was added to BEAS-2B cells. For co-infections, PAO1 at MOI 10 and one commensal at MOI 10 were added to BEAS-2B cells. Gentamicin (125 µg/ml) was added 2 h post-infection to kill the bacteria and the plates were then incubated overnight at 37°C, 5% CO_2_. 10µl of the media were thereafter cultured overnight on CBA plates and inhibition of extracellular bacteria by gentamicin at the concentration used was confirmed. The plates were centrifuged and cell culture supernatant was collected for the measurement of inflammatory marker IL-8. For classical PAMP stimulation, BEAS-2B were treated with 1 µg/ml ultrapure LPS derived from *Salmonella minnesota* R595 (*In vivo*Gen, San Diego, CA, USA). To check if there are any differences in *P. aeruginosa* amount after mono-infection and co-infection with different *S. mitis* strains, after 2h infection *P. aeruginosa* in the supernatant was serially diluted and incubated overnight on CBA and then counted.

### Measurement of Cytokine/Chemokine Secretion

Sandwich enzyme-linked immunosorbent assay (ELISA) was performed using commercially available kits (BD Biosciences, human IL-8 ELISA, San Jose, CA, USA) to determine the amount of secreted human IL-8 in the cell-free supernatants of infected cells. The assays were performed according to the manufacturers’ instructions. Cytokine concentrations were calculated using Gen5 V2.09 software (BioTek, Winooski, VT, USA) with 0.8 pg/ml as the lowest detectable value.

The multiplex bead-based assay LEGENDplex Mouse Anti-Virus Response Panel (BioLegend, San Diego, CA, USA) was used to assess cytokine/chemokine levels in conditioned supernatants of infected PCLS according to the manufacturer’s instructions.

### Generation of Precision Cut Lung Slices From Mice

Wild type C57BL/6N male mice (Charles River Laboratories) aged 8-12 weeks were used for the experiment. Mice were housed and bred under specific pathogen-free (SPF) conditions in the animal facility of the TU Dresden. PCLS were prepared as previously described (Kolbe et al., 2020). In short, mice were scarified by intraperitoneal injection with 240 mg/kg ketamine (Bremer Pharma, Warburg, Germany) and 32 mg/kg xylazine (CP-Pharma, Burgdorf, Germany) in 0.9% sodium chloride solution, and then the lungs were inflated with 0.035 ml/g bodyweight of a 20% gelatin suspension in sterile water (Aqua ad iniectabilia Braun, B. Braun, Melsungen, Germany). Subsequently, the left lung lobe was excised, rinsed with PBS, and gelatin-embedded in a cubic mold on ice. Upon solidification, lungs were cut transversely into 250 µm slices using a Leica Vibratome VT1200 or VT1200S (Wetzlar, Germany). PCLS were collected after discarding the top quarter of the lobe and placed into DMEM/F12 containing 15 mM HEPES, 2.5 mM L-glutamine (Gibco, Darmstadt, Germany), supplemented with 1% Penicillin/Streptomycin (Gibco, Darmstadt, Germany), in a 24-well plate on ice. Two randomly chosen PCLS were cultured per well. After completion of the cutting, culture medium was replaced by 500 µl DMEM/F12 supplemented with 1% Penicillin/Streptomycin and PCLS were maintained at 37°C, 5% CO2, and 95% humidity. The medium was exchanged over 3h to prevent contaminations and to remove the resolved gelatin. Generation of PCLS was considered to be completed afterwards. Experiments were done according to the local animal welfare rules and upon notification of the local authorities.

### Infection of Precision Cut Lung Slices

Infection of PCLS was carried out as previously described (Kolbe et al., 2020) with slight modifications. Briefly, one day post cutting, the overnight incubation medium was replaced by DMEM/F12 (Gibco, Darmstadt, Germany) before conducting experiments. Bacteria were grown on CBA plates overnight at 37°C and then harvested and suspended in DMEM/F12 (Gibco, Darmstadt, Germany) with concentration being adjusted according to McFarland measurement based on the corresponding CFU count. PCLS were infected with either *P. aeruginosa* PAO1 suspension, or *Streptococcus mitis* strain SM4 suspension, or PAO1/SM4 mixed bacterial suspension (the CFU ratio of PAO1:SM4 is 1:6). The CFUs of PAO1(4 x 10^6^ CFU/ml) or SM4 (2.4 x 10^7^ CFU/ml) used for mono- or co-infection were equal. The CFUs of PAO1 were chosen based on a previous publication (Kolbe et al., 2020) that this range of *P. aeruginosa* amount is able to trigger cytokine responses in PCLS. PCLS were either subjected to RNA isolation eight hours post infection, or 125 µg/ml gentamicin (Carl Roth, Karlsruhe, Germany) was added per well four hours post infection to stop the infection and the samples were incubated further overnight before the collection of supernatants for cytokine/chemokine measurement. 10µl of the media were cultured overnight on CBA plates and inhibition of bacteria by gentamicin at the concentration used was confirmed. To check if there are any differences in *P. aeruginosa* amount after mono-infection and co-infection, after 8h infection, *P. aeruginosa* in the culture media was serially diluted and incubated overnight on CBA and then counted.

### RNA Sequencing and Transcriptome Analysis

For transcriptome analysis, RNA was isolated with the RNeasy Mini Kit (Qiagen, Hilden, Germany) from non-treated PCLS, PCLS mono-infected with *P. aeruginosa* PAO1, PCLS mono-infected with *S. mitis* SM4, and PCLS co-infected with PAO1/SM4 8h post infection. RNA sequencing was performed at the DRESDEN-concept Genome Center at the Center for Molecular and Cellular Bioengineering (CMCB), Technische Universität Dresden, Germany. First, mRNA was enriched with poly-dT pulldown according to the instructions of the NEBNext Poly(A) mRNA Magnetic Isolation Module (New England Biolabs, Ipswich, MA, USA); second, libraries were prepared using the NEBNext Ultra II Directional RNA Library Prep Kit for Illumina (New England Biolabs, Ipswich, MA, USA); third, sequencing was performed on a NovaSeq6000 (Illumina Inc., San Diego, CA, USA) in single-end mode (75 bp). For each sample, around 30 million fragments were sequenced. Resulting sequences were trimmed for quality using Sickle 1.33 (parameters: quality >30; length>45). Raw fastq-files that passed quality control were pseudoaligned to the murine transcriptome model (mm10, GENCODE general assembly release vM16) with the Kallisto pipeline (Bray et al., 2016). Then with the tximport package transcript counts were imported into R, and the information of transcript levels was summarized to a gene-level count table. Further analyses of differentially expressed genes (DEs) and functional enrichment were performed with R package DESeq2 (Love et al., 2014) and ClusterProfiler (Yu et al., 2012; Wu et al., 2021). Genes showing differential expression (|FC| ≥ 1.5, adjusted p-value ≤ 0.05) were used as input for the functional enrichment analysis. R package ggplot2 (version 3.3.0) was used to visualize the data of normalized counts generated by DESeq2. Sequence data is available publicly (https://doi.org/10.6084/m9.figshare.17118539).

### Whole Genome Sequencing of Bacteria

Bacterial DNA was isolated with the DNeasy Blood & Tissue Kit (Qiagen, Hilden, Germany) following the manufacturer’s instructions. Library preparation and sequencing on a MiSeq Illumina platform (short-read sequencing 2x300bp) were performed as previously described (Kocer et al., 2020). In short, raw sequences were controlled for quality using sickle (v1.33, parameter: -q 30 -l 45), assembled with SPAdes 3.13.0 (with the option –careful and –only-assembler). Draft genomes were curated by removing contigs with a length < 1000bp and/or coverage < 10X. The quality of the final draft was quality controlled using Quast (v5.0.2). The draft genome sequences are deposited in the NCBI GenBank database under the project number PRJNA782624. Sequencing statistics are available in the **Supplementary Material** (**Table S1**).

### Whole-Genome Sequence Analyses

Whole-genome comparisons by average nucleotide identity (ANI) was calculated for MUMmer based ANI (ANIm) with PYANI (Pritchard et al., 2016). Pan-genome analysis of *Streptococcus mitis* strains, including estimates for genome completeness based on domain-level single-copy core genes, was performed with software anvi’o (version 7) (Eren et al., 2015; Eren et al., 2021) following the workflow for microbial pangenomics (Delmont and Eren, 2018; Yi and Dalpke, 2022). In detail, PGAP (Tatusova et al., 2016) was used to identify open reading frames, then each database was populated with profile hidden Markov models (HMMs) by comparing to a collection of single-copy genes using HMMER (Eddy, 2011). All genomes were annotated with the NCBI functional annotation source COGs (Clusters of Orthologous Genes) (Galperin et al., 2015; Galperin et al., 2021), which was accomplished by running anvi’o workflow “anvi-run-ncbi-cogs” that uses Diamond (Buchfink et al., 2015) to search the NCBI COG database and then annotates genomes accordingly. We used the Diamond software to calculate gene similarity and MCL (van Dongen and Abreu-Goodger, 2012) for clustering under the following settings: minbit, 0.5; mcl inflation, 2.0; and minimum occurrence, 1.

### Statistical Analysis

All experiments were performed with at least three independent biological replicates. To compare one variable between more than two groups, we performed One-way-ANOVA with Sidak as post-test to correct for multiple comparisons. Significant results are indicated with stars as: *p < 0.05; **p < 0.01; ***p < 0.001; ****p <0.0001. GraphPad Prism version 8 for Windows (GraphPad Software, San Diego, CA, USA, www.graphpad.com) was used for statistical analysis and visualization.

## Results

### Isolation of a Collection of Aerobic and Facultative Anaerobic Commensal Strains From Cystic Fibrosis Microbiome Samples

Based on previous findings from CF microbiome studies [e.g. (Boutin et al., 2017; Zemanick et al., 2017; Frey et al., 2021)], the hypothesis was raised that commensal bacteria in the CF airway microbiome might have protective effects against inflammation. The aim of this study was to isolate commensal bacteria from CF patients and to test them *in vitro* for their hypothetical abilities of interference with *P. aeruginosa* mediated inflammation. Therefore, bacterial species that were not considered to be classical pathogens and recognized as part of the commensal airway microbiota according to literature information [e.g. (Man et al., 2017; de Steenhuijsen Piters et al., 2020)] were isolated from CF sputum samples, and we have acquired a collection of 83 aerobic or facultative anaerobic commensal bacterial strains (**Table S2**). Part of the collection was commensals from sputa of CF patients that were documented being negative for *P. aeruginosa* and that were also part of a clinical study (Frey et al., 2021). A fraction of the collection was commensals isolated from sputa of CF patients for which the infection status with *P. aeruginosa* at the exact time of sampling was not documented. As listed in **Table S2**, isolated strains were from genera *Streptococcus*, *Neisseria*, *Actinomyces*, *Corynebacterium*, *Dermabacter*, *Micrococcus* and *Rothia*. From each genus, 1-10 species were identified. From each species, usually multiple strains were isolated. Bacterial strains in this study mostly refer to isolates from different subjects. Occasionally if one isolate shows various phenotypes, that isolate was further separated based on phenotype difference. *Streptococcus* strains were most abundantly encountered and included in the collection in a great variety.

### Screening of Commensal Isolates to Identify Strains That Reduce *P. aeruginosa* Triggered Inflammatory Responses

**Figure 1:**
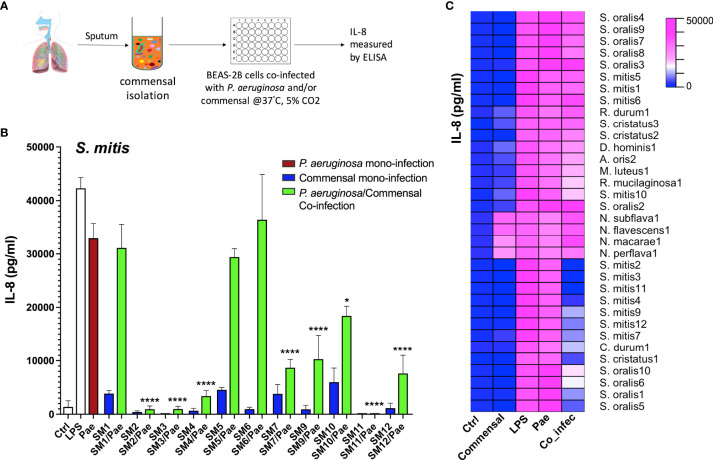
Multiple *Streptococcus* strains reduce *P. aeruginosa* triggered inflammatory responses as indicated by the reduction of IL-8 in co-infection compared to that of *P. aeruginosa* mono-infection in BEAS-2B cells. **(A)** Cell culture based experimental workflow of the screening study. (B&C) BEAS-2B cells were non-infected (Ctrl), or mono-infected with *P. aeruginosa* PAO1 (labelled as Pae) or one CF-microbiome isolated commensal strain, or co-infected with both of them. LPS stimulation is used as positive control. Release of IL-8 into the supernatant was assessed after 2 h of infection followed by overnight incubation. **(B)** Representative results from *Streptococcus mitis* strains (“SM+number” is the name of self-isolated strains). Data are presented as Mean ± SEM (N=6 experiments). *p < 0.05, ****p < 0.0001 indicate significant difference between co-infection and *P. aeruginosa* mono-infection. **(C)** The major results are summarized as a heatmap. Ctrl, non-infected control samples; Commensal, commensal mono-infection; LPS, LPS stimulation as positive control; Pae, *P. aeruginosa* PAO1 mono-infection; Co_infec, *P. aeruginosa*/commensal co-infection. S., *Streptococcus*; N., *Neisseria*; D., *Dermabacter*; A., *Actinomyces*; M., *Micrococcus*; R., *Rothia*; C., *Corynebacterium.* Strain name such as SM4 is labelled as S. mitis4; N=6 experiments.

In order to analyze whether certain commensal strains isolated from CF airway microbiome are able to reduce *P. aeruginosa* triggered inflammatory responses, we performed a screening study using human airway epithelial cells BEAS-2B with the workflow shown in **Figure 1A**. Massive production of the proinflammatory Interleukin (IL)-8 is one of the major characteristics associated with severe lung inflammation in CF and plays an important role in CF lung pathophysiology (Corvol et al., 2003; Montemurro et al., 2012). Thus, we chose IL-8 as a marker cytokine to measure the extent of inflammatory responses. BEAS-2B cells were mono-infected with one commensal strain or *P. aeruginosa* (PAO1), or co-infected with commensal/*P. aeruginosa* combinations. Representative results from *Streptococcus mitis* strains are shown in **Figure 1B**, and the major results are summarized as a heatmap in **Figure 1C**. More detailed information of all strains tested is listed in **Table S3**. From the 83 collected commensal bacteria, 80 could be tested. The results revealed that various *Streptococcus* strains, especially several strains belonging to the species *S. mitis*, *S. oralis* and *S. cristatus* showed strong inhibitory effects on *P. aeruginosa* triggered inflammatory responses, indicated by a significant reduction of IL-8 production in co-infection compared to that of *P. aeruginosa* mono-infection. In particular, the co-infection of a few strains from *S. mitis* (SM2, SM3, SM4 and SM11) with *P. aeruginosa* led to more than 9-fold reductions (p<0.0001) of IL-8 compared to that of *P. aeruginosa* mono-infection, indicating protective effects of these *S. mitis* strains on the host.

We also observed remarkable intra-species differences. Multiple *S. mitis* strains, e.g. SM1, SM5 and SM6 showed no protective effects. No differences in *P. aeruginosa* amount were detected after 2 h co-infection with different *S. mitis* strains with or without protective effects (**Supplementary Material Figure S1**), indicating the reduction of IL-8 production was not derived from early inhibition on the growth of *P. aeruginosa*. Different from *Streptococcus* strains, strains from most other genera rarely showed protective effects, such as *Neisseria* (**Figure 1C**).

### Commensal Bacteria Lower the *P. aeruginosa* Triggered Immune Stimulation in the *Ex-Vivo* System PCLS

**Figure 2:**
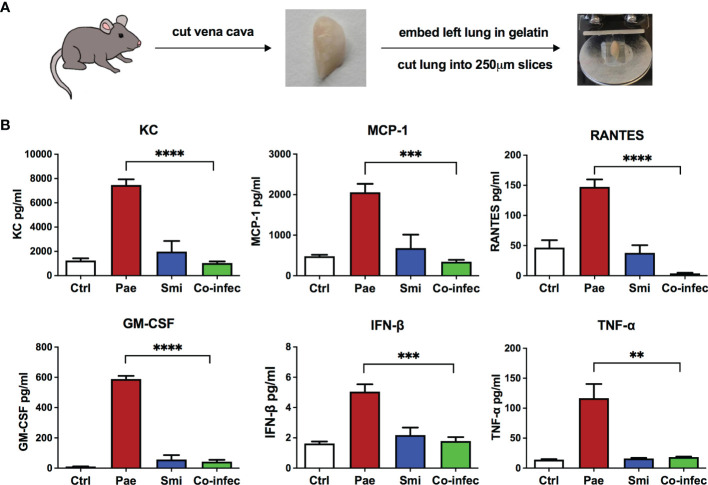
Cytokine induction in response to mono-infection of *P. aeruginosa* or *S. mitis* or co-infection of them in mouse PCLS. **(A)** A brief scheme of PCLS generation workflow. **(B)** PCLS were non-infected (Ctrl) or mono-infected with *P. aeruginosa* PAO1 (Pae), *S. mitis* SM4 (Smi) or co-infected with both of them (Co-infec). Release of several cytokines into the supernatant was assessed after 4 h of infection followed by overnight incubation. Data are presented as Mean ± SEM of three independent experiments. * indicate significant difference between co-infection and *P. aeruginosa* mono-infection. **p < 0.01, ***p < 0.001, ****p < 0.0001.

*P. aeruginosa*/commensal co-infection experiments in airway epithelial cells BEAS-2B showed that several commensal strains, especially strains belonging to *S. mitis*, reduced *P. aeruginosa* triggered inflammatory response. To further verify the results from BEAS-2B cells, we conducted infection experiments with an *ex vivo* lung model system - murine precision cut lung slices (PCLS). PCLS can be used to investigate the regulation of immune responses as a complex interplay between pulmonary cells organized in their natural architecture rather than an isolated pulmonary cell type (Kolbe et al., 2020). For PCLS generation, gelatin-inflated lungs of euthanized mice were isolated, then the left lobe was separated, gelatin-embedded, and cut into thin slices, PCLS (**Figure 2A**). We set out to analyze their response to *P. aeruginosa* mono-infection, *S. mitis* mono-infection and *P. aeruginosa/S. mitis* co-infection.

Protective effects mediated by co-infection were examined using *P. aeruginosa* (PAO1) and one selected *S. mitis* strain SM4, which in BEAS-2B cells showed strong inhibition on *P. aeruginosa* triggered inflammatory responses. We analyzed the secretion of multiple cytokines/chemokines using a cytokine bead assay, and we observed reduced KC, MCP-1, RANTES, GM-CSF, IFN-β and TNF-α secretion in co-infection compared to *P. aeruginosa* mono-infection (**Figure 2B**). The decreased secretion of cytokines/chemokines in co-infection indicated that in PCLS the *S. mitis* strain SM4 could also inhibit *P. aeruginosa* triggered inflammatory responses thus confirming the findings with BEAS-2B cells. We also verified that the inhibitory effects were not derived from reduction of *P. aeruginosa* growth: *P. aeruginosa* amounts after 8h mono-infection or co-infection with SM4 in the PCLS showed no differences (**Figure S2**).

### Signaling Pathways Involved in Mediating Inflammatory Responses Are Downregulated by Co-Infection

**Figure 3:**
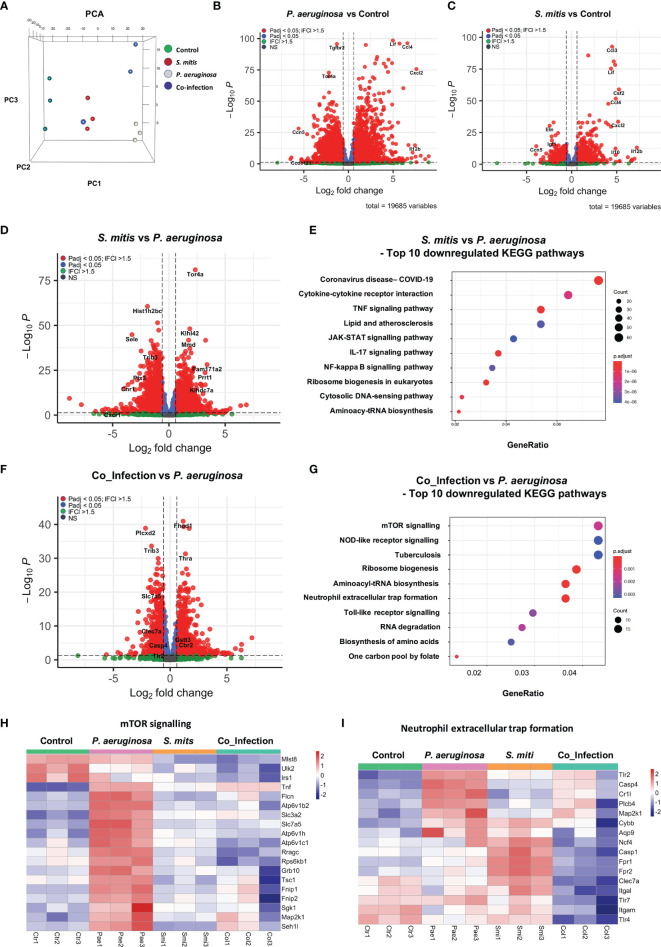
Signaling pathways involved in mediating inflammatory responses are downregulated in co-infection compared to *P. aeruginosa* mono-infection in mouse PCLS. **(A)** 3D PCA plot of three independent experiments. Non-treated PCLS (Control) and PCLS infected with *P. aeruginosa* or *S. mitis*, or co-infected with both two bacteria (Co-infection) were lysed 8 h post infection for RNA isolation and expression profiling. **(B–D, F)** Volcano Plot of genes that are significantly different between the comparison “*P. aeruginosa* vs. Control” **(B)**, “*S. mitis* vs. Control” **(C)**, “*S. mitis* vs. *P. aeruginosa*” **(D)**, “Co_infection vs. *P. aeruginosa*” **(F)**. FC, fold change; Padj, p-value adjusted; NS, not significant. **(E)** Signaling pathway analysis was performed with genes downregulated in the comparison “*S. mitis* vs. *P. aeruginosa*” (fold change ≤−1.5 and p-value adjusted ≤0.05). Top 10 downregulated KEGG pathways are displayed. **(G–I)** Signaling pathway analysis was performed with genes downregulated in the comparison “Co_infection vs. *P. aeruginosa*” (fold change ≤−1.5 and p-value adjusted ≤0.05). **(G)** Top 10 downregulated KEGG pathways. **(H)** Heatmap of genes grouping into the KEGG pathway “mTOR signaling”. **(I)** Heatmap of genes grouping into the KEGG pathway “Neutrophil extracellular trap formation”. The genes shown in the heatmaps fulfill the criteria: fold change ≤−1.5 and p-value adjusted ≤0.05. Ctr, non-treated PCLS; Pae, *P. aeruginosa* mono-infected PCLS; Smi, *S. mitis* mono-infected PCLS; Col, *P. aeruginosa* and *S. mitis* co-infected PCLS.

To investigate the mechanisms by which selected commensal bacteria reduce *P. aeruginosa* triggered inflammatory responses, we performed RNA expression profiling comparing non-treated PCLS (Control), PCLS mono-infected with *P. aeruginosa* or *S. mitis* (PAO1 and SM4 are the corresponding strains used in the transcriptome analysis), and PCLS co-infected with *P. aeruginosa* and *S. mitis*. Principal component analysis (PCA) showed a distinct separation of all four groups. *S. mitis* mono-infected and non-treated samples were closer to each other compared to other groups, whereas *P. aeruginosa* mono-infected samples showed a prominent separation (**Figure 3A**). Accordingly, we found that 5,852 genes were significantly regulated in PCLS upon *P. aeruginosa* mono-infection compared to non-treated PCLS (*P. aeruginosa vs* Control in **Figure 3B**; top 50 up-regulated and down-regulated genes are listed in **Tables S4, S5**). In contrast, *S. mitis* mono-infection significantly regulated only 1,796 genes compared to non-treated PCLS (*S. mitis vs* Control in **Figure 3C**; top 50 up-regulated and down-regulated genes are listed in **Tables S6, S7**).

The huge number of differentially regulated genes illustrates that *P. aeruginosa* triggered profound rewiring of the PCLS transcriptome whereas *S. mitis* was much less active. Through direct comparison of the transcriptome profiles in response to *S. mitis* mono-infection and *P. aeruginosa* mono-infection (*S. mitis* vs *P. aeruginosa* in **Figure 3D**; top 50 up-regulated and down-regulated genes are listed in **Tables S8, S9**), we found that 1,957 genes were expressed at a much lower level in response to *S. mitis* mono-infection compared to that of *P. aeruginosa* mono-infection. Signaling pathway analysis indicated that most of these genes are implicated in immune responses (e.g. TNF signaling and IL-17 signaling) (**Figure 3E**).

Furthermore, we found that 2,796 genes were differentially expressed between PCLS co-infected by *P. aeruginosa*/*S. mitis* and PCLS mono-infected by *P. aeruginosa* (Co_Infection *vs P. aeruginosa* in **Figure 3F**; top 50 up-regulated and down-regulated genes are listed in **Tables S10, S11**), highlighting a considerable difference in PCLS response to co-infection compared to *P. aeruginosa* mono-infection. Pathway analysis revealed that co-infection of PCLS turned down inflammatory responses triggered by *P. aeruginosa* mono-infection as seen by the down-regulation of multiple signaling pathways that are involved in mediating inflammatory responses, such as mTOR signaling pathway, NOD-like receptor signaling, and Neutrophil extracellular trap formation (**Figure 3G**). Detailed pathway analysis of mTOR signaling (**Figure 3H**) and neutrophil extracellular trap formation (**Figure 3I**) revealed the down-regulated genes that are associated with these two signaling pathways. Multiple genes in these two gene lists are known playing an important role in mediating inflammatory response, such as TSC1 (Zhu et al., 2014) and TLR2 (Oliveira-Nascimento et al., 2012; Fereig et al., 2019). We also compared PCLS co-infected by *P. aeruginosa*/*S. mitis* and PCLS mono-infected by *S. mitis*, and 1386 genes showed differential expression (top 50 up-regulated and down-regulated genes are listed in **Tables S12, S13**).

### Whole-Genome Sequence Comparison Provides Insights Into Underlying Mechanism of the Protective Effects

**Figure 4:**
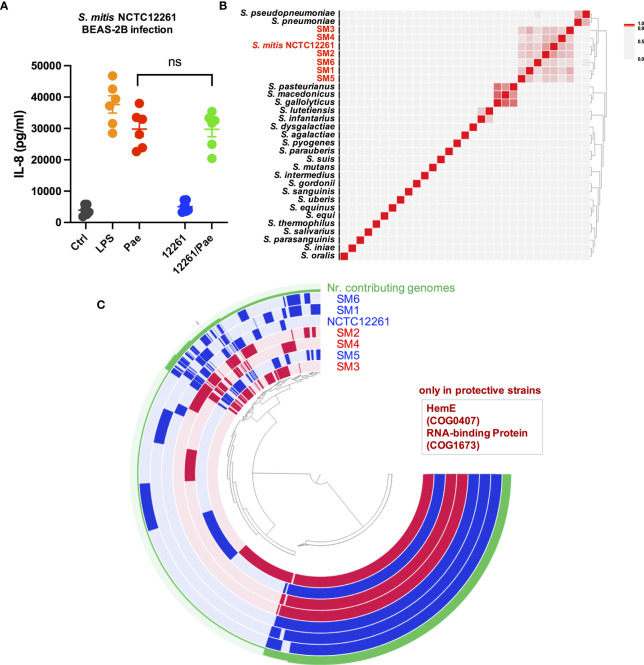
Pan-genome analysis reveals genes with specific functions only present in the protective strains SM2, SM3 and SM4. **(A)** Co-infection of *S. mitis* type strain NCTC12261(labelled as 12261) and *P. aeruginosa* PAO1 (Pae) in BEAS-2B cells could not reduce the IL-8 production triggered by *P. aeruginosa* infection. ns, not significant. **(B)** ANIm analysis of complete whole genome sequences of *Streptococcus* type strains with SM1, SM2, SM3, SM4, SM5 and SM6. A heatmap and dendrogram of ANIm between these genomes is shown. **(C)** Pan-genome analysis to compare strains with protective effects and strains without protective effects. One layer represents one genome, with one extra layer showing number of contributing genomes to each orthologous gene family (Nr. contributing genomes). Genomes of strains showing protective effects (SM2, SM3, SM4) are colored in red, and genomes of strains without protective effects (NCTC12261, SM1, SM5 and SM6) are colored in blue. The clustering is based on presence/absence of orthologous gene family using Euclidean distance and Ward Linkage. Two genes with function annotation COG0407 and COG1673 are only detected in strains with protective effects, while missing in strains without protective effects.

To further investigate the underlying mechanism of the protective effects at the bacterial genome level, we thought to make use of the observation that within some commensal species only a couple of strains showed protective effects, thus making it possible to identify genetic elements only present in the strains with protective effects while missing in the strains without protective effects. Owing to the remarkable intra-species differences among *S. mitis* strains, a first study with several selected *S. mitis* strains was done. The strains SM2, SM3 and SM4 showed strong protective effects in co-infection experiments, so we selected SM2, SM3 and SM4 as the protective strains. The strain SM1, SM5 and SM6 showed no protective effect in the co-infection experiment, so we chose SM1, SM5 and SM6 as the ‘protective effect negative’ strain. Whole genome sequencing was performed for SM1, SM2, SM3, SM4, SM5 and SM6. The whole genome sequence of *S. mitis* type strain NCTC12261 is publicly available (NCBI assembly accession: GCF_000148585.2). However, we did not know if NCTC12261 has protective effects or not, so we performed a co-infection experiment with NCTC12261 in BEAS-2B cells. The results indicate NCTC12261 could not reduce the *P. aeruginosa* triggered inflammatory responses (**Figure 4A**). This means NCTC12261 can be counted as an ‘protective effect negative’ strain together with SM1, SM5 and SM6.

By whole genome sequence comparison through ANIm analysis, we first compared the SM1, SM2, SM3, SM4, SM5 and SM6 sequences with publicly available whole genome sequences from type strains belonging to the genus *Streptococcus*, including the genome sequence from *S. mitis* NCTC12261 (sequence information of the type strains is listed in **Table S14**). Among all the type strains, these six CF microbiome isolated *S. mitis* strains showed the highest ANIm value when paired with *S. mitis* NCTC12261, and the seven strains were closely clustered together as shown in **Figure 4B**, supporting their correct taxonomy as *S. mitis*.

We then compared several important parameters of the whole genome sequences of the seven strains: SM1, SM2, SM3, SM4, SM5, SM6 and NCTC12261 (**Table S15**). Genome completion of the protective strains SM2, SM3 and SM4 is 100%, 100% and 99%, respectively, indicating the genome sequences are complete or almost complete. The four negative strains SM1, SM5, SM6 and NCTC12261 also have a high genome completion of 100%, 96%, 100% and 100%, respectively. Therefore, we could compare the whole genome sequences of these strains to search for functions that are associated with the protective effects by identifying functions only present in SM2, SM3 and SM4 (based on function annotation from the NCBI COG database COG20). For that, we conducted pan-genome analysis of SM1, SM2, SM3, SM4, SM5, SM6 and NCTC12261 (**Figure 4C**). Since different proteins can be characterized with the same COG function (Tatusov et al., 2003; Galperin et al., 2021), through pan-genome analysis we aim to identify proteins with COG functions only present in the protective strains SM2, SM3 and SM4. The results of pan-genome analysis reveals two genes with specific functions only present in the protective strains SM2, SM3 and SM4, while missing in the ‘protective effect negative strains’ SM1, SM5, SM6 and NCTC12261 (labelled as “only in protective strains”): 1. Uroporphyrinogen-III decarboxylase HemE (HemE) (COG0407; PDB:2DG5), which belongs to the COG20 pathway: Heme biosynthesis, and belongs to COG20 category H; 2. one predicted RNA-binding protein, contains PUA-like EVE domain (COG1673; PDB:1WMM), which belongs to COG20 category R. We have further verified the results with whole genome sequence of SM11, which also shows strong protective effects in BEAS-2B co-infection experiments, and genome sequence analyses have revealed that these two genes are present in SM11 as well.

## Discussion

*P. aeruginosa* may cause destructive chronic pulmonary infections in people suffering from CF (Murray et al., 2007; Jonckheere et al., 2018). In many people with CF, the airways are inhabited by various species of microorganisms, and oral commensal Streptococci are frequently detected habitants of the CF lung. However, only a limited number of studies have been performed so far to investigate the interplay between *P. aeruginosa* and Streptococci, and the results of these studies indicate that certain Streptococci might be able to potentiate *P. aeruginosa* infection (Whiley et al., 2014; Waite et al., 2017), while some other Streptococci may attenuate the pathogenesis of *P. aeruginosa* in the CF lung (Scoffield and Wu, 2016; Scoffield et al., 2017; Tuncer and Karacam, 2020). The exact effect seems to be species or strain specific (Whiley et al., 2015). Furthermore, few studies have explored the host-pathogen-commensal interactions.

In this study, through screening of commensal bacterial strains isolated from CF airway microbiome samples, multiple commensal strains that may reduce *P. aeruginosa* triggered inflammatory responses have been identified, and many of those belong to the genus *Streptococcus*. The protective effects of *S. mitis* strains were examined with human airway epithelial cells BEAS-2B, and further verified with *ex vivo* system mouse PCLS representing small functional pulmonary units. The underlying mechanisms for the complex host-pathogen-commensal interactions were investigated from the host side and also from the bacterial side. Transcriptome changes in the host in response to co-infection and mono-infection were evaluated by RNA deep sequencing, and the genomic differences among bacterial strains with and without protective effects were investigated by whole genome sequencing. Transcriptomic analysis comparing mono- and co-infection indicated that several signalling pathways involved in mediating inflammatory responses were downregulated in co-infection compared to *P. aeruginosa* mono-infection, such as neutrophil extracellular trap formation. The results of whole genome sequence comparison of strains with and without protective effects revealed two genes only present in the strains showing protective effects. One gene encodes a predicted RNA-binding protein, which contains PUA-like EVE domain, and the other one encodes Uroporphyrinogen-III decarboxylase HemE (HemE), which plays an important role in heme biogenesis.

For the cell infection experiments in this study, the dose used (MOI 10) is clinically relevant. A total bacterial MOI of about 100 has been reported for CF patients (Sze et al., 2012), and the relative abundance of *Streptococcus* in people with CF varies, with a lowest detectable abundance of 0.01% and a highest abundance of 87.05% being reported (Zemanick et al., 2017), supporting the clinical relevance of the dose used. The CFUs of bacteria used in PCLS infection (*P. aeruginosa*: approx. 4 x 10^6^ CFU/ml; *Streptococcus*: approx. 2.4 x 10^7^ CFU/ml) are also clinically relevant. It is known that in microbiome samples from people with CF, viable total bacterial densities can range from 3.5 × 10^6^ CFU/ml to 5.9 × 10^12^ CFU/ml, and viable *P*. *aeruginosa* densities range from 5.3 × 10^3^ CFU/ml to 1.8 × 10^11^ CFU/ml (Stressmann et al., 2011). In the *ex vivo* model used (PCLS), a higher amount of *Streptococcus* compared to *P. aeruginosa* was needed to obtain an anti-inflammatory effect, which is the reason for the CFU ratio chosen in this study. This is corresponding to observation from one previous CF study that the relative abundance of *Streptococcus* was inversely associated with airway inflammation, when controlling for *Pseudomonas* relative abundance (Zemanick et al., 2017).

These findings about the protective effects of *S. mitis* in the CF microbiome are consistent with various previous reports. A few CF studies originating from airway microbiome sequencing indicate that the presence of several commensal bacteria is positively associated with better lung function and less severe disease burden (Coburn et al., 2015; Flight et al., 2015; Acosta et al., 2018; Muhlebach et al., 2018; Ronan et al., 2018; Yi et al., 2021), indicating that the presence of commensal bacteria might be one essential factor for shaping healthy lung microbiome and inhibiting disease progression related to *P. aeruginosa* infection. It is also noteworthy that in one clinical CF study it has been shown that prevalence of Streptococci in the patient sputum samples is associated with patient stability (Filkins et al., 2012). Furthermore, in a recent publication of our own (Frey et al., 2021), we could show that in CF patients, a more diverse sputum microbiome was associated with better lung function and less inflammation, and the microbiome of patients with less inflammation typically have various commensal airway bacteria, including *Streptococcus*, indicating that *Streptococcus* may possibly interfere with *P. aeruginosa* mediated inflammation. It has also been reported by another CF microbiome study that the relative abundance of *Streptococcus* was inversely associated with airway inflammation in CF patients (Zemanick et al., 2017).

However, the exact role of those commensals remains poorly understood. Furthermore, most of the studies so far focused on the bacterial pathogen part, and the counterpart of the host response was unknown or only measured with a low number of immunological readouts. It has become clear that CF lung disease is associated with dysregulation of host defense systems, finally disrupting the balance between inflammation and resolution. This disruption results in host susceptibility to infection with neutrophils and macrophages playing important roles (Cohen-Cymberknoh et al., 2013; Stoltz et al., 2015; Leveque et al., 2017). Although host responses to classical CF pathogens have been well studied, host responses to the polymicrobial nature of CF airway infections remain unclear. In our study, the results of host transcriptomic profiling indicated that the interaction between *S. mitis* and *P. aeruginosa* could down-regulate various signaling pathways mediating inflammatory responses in the host, and several of these signaling pathways are implicated in the pathogenesis of CF. In particular, the role of neutrophil extracellular trap (NET) formation in CF pathogenesis has been studied in detail. The excessive formation of NETs contributes to chronic infection of certain opportunistic pathogens such as *P. aeruginosa* and host tissue damage in CF (Young et al., 2011; Manzenreiter et al., 2012; Saffarzadeh et al., 2012), and often leads to a vicious cycle of bacterial colonization, airway inflammation, and lung structure damage, which may cause irreversible lung damage and decline of lung function in people with CF (Hartl et al., 2012). The reduction of NET formation in co-infection in PCLS is also consistent with the results from the human BEAS-2B cells since excessive NET formation in CF pathogenesis can be induced by high level of IL-8 in humans (Brinkmann et al., 2004), while reduction of IL-8 may decrease NET formation accordingly.

Importantly, we observed that there was no difference in *P. aeruginosa* amount after 2 h co-infection with different *S. mitis* strains with or without protective effects, indicating the reduction of host inflammatory responses was not derived from early inhibition on the growth of *P. aeruginosa.* We assume the modulation of host immune responses by the pathogen-commensal interaction might be achieved *via* either a direct impact on host immune responses, or an indirect impact on *P. aeruginosa* pathogenicity through modification of micro-environment by metabolism adjustment, or both play a role in mediating the protective effects.

The underlying mechanism was further investigated with whole-genome sequence comparison, and we have found two genes only present in the strains that may reduce inflammation. One gene encodes a predicted RNA-binding protein with PUA-like EVE domain, and it´s functional role has not been investigated yet. Based on literature information, our speculation is that this RNA-binding protein can modify protein expression level and accordingly adjust bacterial metabolism and modify micro-environment. Interestingly, the presence of Uroporphyrinogen-III decarboxylase HemE in the strains with protective effects corresponds to a few previous discoveries. As one important iron source, heme is utilized by both eukaryotes and prokaryotes for critical cellular processes, such as DNA synthesis and cellular respiration (Frankenberg et al., 2003). During bacterial pathogen infection, Gram-negative pathogens have various mechanisms to obtain iron from heme to maintain fitness. However, at the same time, Gram-negative pathogens must strictly regulate the intracellular concentrations of iron and heme, because high levels of iron can lead to the production of harmful reactive oxygen species (Choby and Skaar, 2016; Hardison et al., 2018; Richard et al., 2019). The expression of HemE in commensal Streptococci might be able to affect heme metabolism of *P. aeruginosa* living in the same environment and therefore impact the pathogenicity of *P. aeruginosa.*

Besides, we cannot rule out the possibility that the interaction might have a direct impact on the host immune responses through certain cell-surface receptors. It is known that airway epithelial cells have innate sensor functions and may detect microbial signals (Weitnauer et al., 2016). It was shown in a recent report that *Rothia mucilaginosa* could inhibit host NF-κB pathway activation in a human lung epithelial cell line (Rigauts et al., 2021), and another commensal *Prevotella histicola* was reported to be able to induce alternative NF-κB activation in CF bronchial epithelial cells (Bertelsen et al., 2020). Earlier work from us has shown that airway epithelial cells adjust their expression pattern of innate immune receptors with less TLR2 and CD36 being expressed, resulting in low reactivity towards microbes that preferentially activate through TLR1/2 or TLR2/6. Gram-positive Streptococci might be mainly sensed through those receptors and as a result these commensal bacteria show no or low immune stimulation (Mayer et al., 2007). This suggests at non-sterile mucosal surfaces, evolution might have resulted in adaptation of microbial sensing mechanism towards tolerance of the normal level of microbial colonization, which might also promote a protective effect inhibiting inflammatory responses triggered by pathogenic bacteria.

Furthermore, it is possible that different commensal strains might have different mechanisms for the protective effects. In future investigations, we would also investigate mechanisms of strains from other species, e.g. *S. cristatus* or species from other genera to get better understanding about the host-pathogen-commensal interactions in the CF context. We have so far only used *P. aeruginosa* lab strain PAO1 in this study. Modulation of inflammation related with *P. aeruginosa* clinical strains can be different. However, preliminary data indicate that inflammatory responses triggered by certain *P. aeruginosa* clinical isolates [e.g. *P. aeruginosa* strain CHA (Toussaint et al., 1993)] can also be inhibited by *S. mitis* (data not shown). This limitation of the current study will be addressed in future investigations.

In summary, through a screening study of commensal bacterial strains isolated from CF microbiome samples, we could identify multiple commensal strains that may strongly reduce host inflammatory responses in co-infection with *P. aeruginosa* compared to that of *P. aeruginosa* mono-infection, and the protective effects are strain-specific. Further mechanistic analyses with a focus on *S. mitis* indicate that the pathogen-commensal interaction could modulate host immune responses and reduce signaling pathways involved in the pathogenesis of CF, which is possibly achieved *via* an indirect impact on *P. aeruginosa* pathogenicity through modification of micro-environment by metabolism adjustment, and/or a direct impact on host immune responses. Research to investigate the exact regulatory network based on the information achieved through this study will be necessary to further understand the protective potential of CF commensals, and enable the development of novel *P. aeruginosa* infection mitigation or prevention strategies in the context of CF microbiome.

## Data Availability Statement

The datasets presented in this study can be found in online repositories. The names of the repository/repositories and accession number(s) can be found below: Genbank: PRJNA782624; Figshare: https://doi.org/10.6084/m9.figshare.17118539.

## Ethics Statement

The animal study was reviewed and approved by Technische Universität Dresden.

## Author Contributions

AT-O isolated bacterial strains and performed BEAS-2B experiments. LW and AT-O performed mouse PCLS experiments. BY conducted the bioinformatic analysis of the transcriptome data. BY and SB performed whole genome sequence analyses. Transcriptome-related graphics were generated by BY. AD, SB, and BY designed, supported, and supervised the study. AT-O, AD, and BY prepared the first draft of the manuscript. All authors contributed to the article and approved the submitted version.

## Funding

This work was supported in part by the German Ministry for Education and Research (82DZL004A1 to SB) and from the German Cystic Fibrosis Association Mukoviszidose e. V. (Project number 1805 to AD and SB).

## Conflict of Interest

The authors declare that the research was conducted in the absence of any commercial or financial relationships that could be construed as a potential conflict of interest.

## Publisher’s Note

All claims expressed in this article are solely those of the authors and do not necessarily represent those of their affiliated organizations, or those of the publisher, the editors and the reviewers. Any product that may be evaluated in this article, or claim that may be made by its manufacturer, is not guaranteed or endorsed by the publisher.
