# Supplementary material for: Commensal Bacteria in the Cystic Fibrosis Airway Microbiome Reduce P. aeruginosa Induced Inflammation
Source: Front Cell Infect Microbiol. 2022 Jan 31;12:824101. doi: 10.3389/fcimb.2022.824101 (PMC8842722; doi:10.3389/fcimb.2022.824101)
Supplement: Supplementary file 1 [file DataSheet_1.pdf]

frontiers

Supplementary Material

1 Supplementary Figures and Tables

1.1 Supplementary Figures

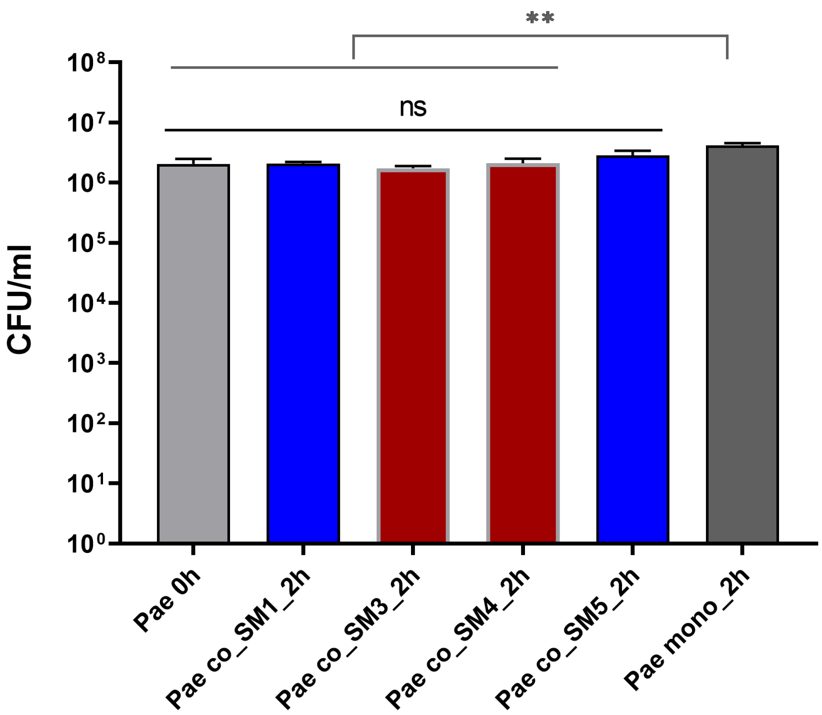

Figure S1: No differences in *P. aeruginosa* amount were detected after 2h co-infection with different *S. mitis* strains with or without protective effects.

SM3&SM4 are strains with protective effects; SM1&SM5 are strains without protective effects. Data are presented as Mean ± SEM (N=6 experiments). ns, no significant difference, ** $p < 0.01$, one-way ANOVA.

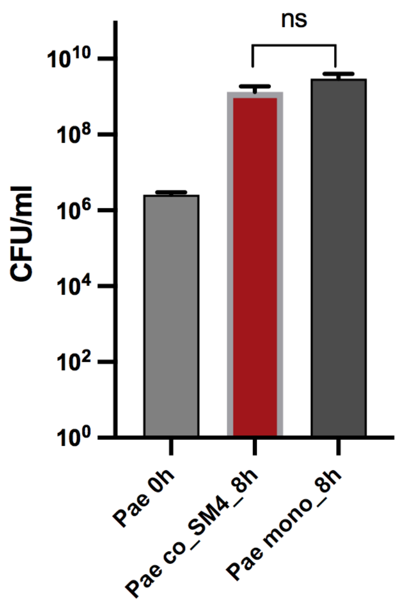

Figure S2: No differences in *P. aeruginosa* amount were detected after 8h mono or co-infection with SM4 in PCLS system.

Data are presented as Mean ± SEM (N=5 experiments). ns, no significant difference.

1.2 Supplementary Tables

Table S1: Genome sequencing statistics.

Isolate	coverage	# contigs	Largest contig	Total length	GC (%)	N50	N75	L50	L75
**SM1**	110	20	552068	2022463	39.76	214308	135863	3	6
**SM2**	39	7	963157	1999481	39.96	587648	587648	2	2
**SM3**	47	225	94075	2827339	40.71	24559	11409	35	79
**SM4**	136	33	245592	1874967	40.28	124961	83053	6	10
**SM5**	67	55	260770	3517890	39.58	137537	65207	9	19
**SM6**	47	34	278851	2012296	39.96	86839	71284	7	13
**SM11**	58	30	708974	1858685	40.44	163595	85870	3	6

Table S2: Overview of the aerobic or facultative anaerobic commensal strains isolated from cystic fibrosis microbiome samples.

Genus	Species	No. of strains of each species
Streptococcus	*S. cristatus*	3
	S. gordonii	3
	S. intermedius	1
	S. constellatus	1
	S. mitis	11
	S. sanguinis	2
	S. oralis	10
	S. parasanguinis	8
	S. salivarius	9
	S. vestibularis	3
Neisseria	*N. flavescens*	5
	N. macacae	4
	N. perflava	4
	N. subflava	7
Actinomyces	*A. oris*	4
Corynebacterium	*C. durum*	1
	C. pseudodiphtheriticum	1
Dermabacter	*D. hominis*	1
Micrococcus	*M. luteus*	2
Rothia	*R. dentocariosa*	2
	R. mucilaginosa	1

Table S3 Summary of results of cell-culture based screening study.

IL-8 (pg/ml) / Isolates	Ctrl	Commensal	LPS	Pae	Co_infec
S. mitis1	1382	820	42282	32915	31112
S. mitis2	1382	431	42282	32915	943
S. mitis3	1382	200	42282	32915	974
S. mitis4	1382	663	42282	32915	3381
S. mitis5	1382	516	42282	32915	32486
S. mitis6	1382	956	42282	32915	36450
S. mitis7	1382	3806	42282	32915	8662
S. mitis9	1382	924	42282	32915	10259
S. mitis10	1382	5980	42282	32915	18426
S. mitis11	1382	200	42282	32915	200
S. mitis12	1382	1141	42282	32915	7594
S. cristatus1	839	729	32542	31516	4582
S. cristatus2	839	704	32542	31516	26917
S. cristatus3	839	5192	32542	31516	31486
S. oralis1	1693	1569	34904	40107	8789
S. oralis2	1693	3951	34904	40107	49045
S. oralis3	1693	2271	34904	40107	38772
S. oralis4	1693	840	34904	40107	34412
S. oralis5	1693	754	34904	40107	5650
S. oralis6	1693	999	34904	40107	14330
S. oralis7	1693	636	34904	40107	30520
S. oralis8	1693	593	34904	40107	28735
S. oralis9	1693	428	34904	40107	32547
S. oralis10	1693	307	34904	40107	16940
N. flavescens1	2939	31430	31676	26408	33525
N. flavescens2	1022	41116	36470	32504	55791
N. flavescens3	1022	36864	36470	32504	55228
N. flavescens4	1022	32142	36470	32504	59564
N. flavescens5	1022	32623	36470	32504	51607
N. macarae1	2939	23955	31676	26408	36565
N. macarae2	2662	53039	38985	30898	51949
N. macarae3	2662	76222	38985	30898	81584
N. macarae4	2662	49681	38985	30898	85639
N. subflava1	2939	31717	31676	26408	34755
N. subflava2	2662	51813	38985	30898	83363
N. subflava3	2662	75601	38985	30898	91190
N. subflava4	2662	53381	38985	30898	46277
N. subflava5	2662	50762	38985	30898	83583
N. subflava6	2662	27475	38985	30898	65689
N. subflava7	2662	46919	38985	30898	83760
N. perflava1	2939	23437	31676	26408	28510
N. perflava2	2960	57937	37804	32243	86651

**N. perflava3**	2960	59200	37804	32243	73013
**N. perflava4**	2960	39632	37804	32243	74864
**S. vestibularis1**	3252	2637	22871	16593	16016
**S. vestibularis2**	3252	2363	22871	16593	23531
**S. vestibularis3**	3252	2490	22871	16593	17877
**A. oris1**	3163	6245	24809	18273	13749
**A. oris2**	2511	4826	34500	29285	21040
**A. oris3**	3163	1332	24809	18273	6739
**A. oris4**	3163	2627	24809	18273	18130
**C. durum1**	2511	2592	34500	29285	11323
**C. pseudodiphtheriticum1**	2322	2022	20107	26749	13603
**D. hominis1**	2511	6429	34500	29285	23654
**M. luteus1**	2511	3681	34500	29285	19676
**M. luteus2**	3495	3627	19936	26749	26973
**R. durum1**	2511	5748	34500	29285	33472
**R. durum2**	4264	1520	21847	24248	18772
**R. mucilaginosa1**	2511	4209	34500	29285	18007
**S. gordonii1**	3150	3497	29564	38136	29081
**S. gordonii2**	3150	1825	29564	38136	36770
**S. gordonii3**	3150	3934	29564	38136	34914
**S. intermedius1**	2783	1849	23215	37384	46299
**S. constellatus1**	2783	2587	23215	37384	17240
**S. parasanguinis1**	2051	3888	35265	51994	62561
**S. parasanguinis2**	2051	4117	35265	51994	50060
**S. parasanguinis3**	2051	3986	35265	51994	56674
**S. parasanguinis4**	2051	1316	35265	51994	55313
**S. parasanguinis5**	2051	1583	35265	51994	47345
**S. parasanguinis6**	2051	591	35265	51994	43072
**S. parasanguinis7**	2051	3454	35265	51994	52465
**S. parasanguinis8**	2051	515	35265	51994	4628
**S. salivarius2**	4584	4373	57630	62109	26847
**S. salivarius3**	4584	2036	57630	62109	31776
**S. salivarius4**	4584	3598	57630	62109	32292
**S. salivarius5**	4584	3829	57630	62109	16071
**S. salivarius6**	4584	2055	57630	62109	25791
**S. salivarius7**	4584	1864	57630	62109	26095
**S. salivarius9**	4584	1685	57630	62109	53971
**S. salivarius10**	4584	2530	57630	62109	53450

Ctrl, non-infected control samples; Commensal, commensal mono-infection; LPS, LPS stimulation as positive control; Pae, *P. aeruginosa* PAO1 mono-infection; Co_infec, *P. aeruginosa*/commensal co-infection. S., *Streptococcus*; N., *Neisseria*; D., *Dermabacter*; A., *Actinomyces*; M., *Micrococcus*; R., *Rothia*; C., *Corynebacterium.* Strain name such as SM4 is labelled as S. mitis4; Data are presented as Mean (N= 3-6 experiments).

Table S4: *P. aeruginosa* vs Control top 50 up-regulated genes.

	log2FoldChange	pvalue	padj
**Il22**	11.0762187090517	1.31256746174381e-11	1.81947677516322e-10
**Gm6482**	9.15015734369736	3.344961106072e-12	5.04013293556579e-11
**Il17a**	9.13103445211335	4.52796780972661e-15	9.9289236059975e-14
**Ifng**	8.99083204197726	1.12332551462484e-07	8.60729939777474e-07
**Gm8024**	8.5997022007032	3.73543028223485e-05	0.000172939979717003
**Gm3141**	8.09350437818714	3.86071501697304e-08	3.20256149035374e-07
**Csf2**	7.82335776300664	2.89482052355852e-138	1.82180704949283e-134
**Cxcl2**	7.61190125053158	2.1321645743611e-79	2.11869827178619e-76
**Ugt1a10**	7.59653831485089	0.0171029272592129	0.0413819385690043
**Prkcg**	7.52049363530978	2.67343695046343e-07	1.88689680840185e-06
**Il12b**	7.45175383019395	5.52600048302894e-17	1.52606053077274e-15
**Tmem132e**	7.18791665804159	7.5528068745943e-11	9.38138117054871e-10
**Tnf**	7.16651764307085	1.65620736302257e-259	3.12691950138661e-255
**Ctla4**	7.06768219383104	4.31137531718359e-06	2.43781868788338e-05
**Ccl20**	6.71139551050229	3.64753768917476e-17	1.03092083191047e-15
**Ccl4**	6.59671412796439	1.98412962199305e-100	5.35148103760411e-97
**Kdm4dl**	6.59315360722256	1.56351374627587e-05	7.90761841138186e-05
**Gm3591**	6.28670106269523	0.000805023120803949	0.00277351031401068
**Csf3**	6.14364271482285	6.27087383294912e-64	3.81916445051869e-61
**CR974586.1**	6.1241846230432	0.000553231254553052	0.00198838874661367
**Il1f6**	6.03198680035272	7.29962988028061e-06	3.92641060227059e-05
**Gm3642**	5.98877521824517	0.00484743651741086	0.0137396188933669
**Npcd**	5.98243481649712	0.00109451570036496	0.00364645428319932
**Gm7945**	5.93302014223463	0.00228358740340168	0.00704824753575671
**Gm43058**	5.90066579269575	9.69091858211158e-05	0.000410879278756494
**Gm8094**	5.89910531359577	0.000218643139755111	0.000860713611045974
**B230303A05Rik**	5.75877521610813	7.86821839246895e-05	0.00034118503272809 8
**Slc32a1**	5.72342771628347	0.00100343156611483	0.00337817189162768
**Cxcl1**	5.71615230640251	3.83672681367268e-100	9.05467528026753e-97
**Gm13509**	5.71435993411038	0.00327100247150187	0.00971625655474438
**Alms1-ps2**	5.68346089588466	2.81748884805106e-06	1.64943223104508e-05
**Olfr1039**	5.61760693176763	0.000357581876036969	0.00133871620455641
**Cxcl11**	5.55078170938028	8.0852929958395e-36	1.07500233634824e-33
**Gm16434**	5.54663993885055	0.00101474856971936	0.00341262076884602
**Gm6676**	5.54663993885055	0.00101474856971936	0.00341262076884602
**4930555G01Rik**	5.54314767446435	0.00779514025153715	0.0208017311588723
**Gm8862**	5.53312380136746	3.07658056497681e-05	0.000145432751794597
**Gp1bb**	5.53010139478322	0.00707862872267257	0.0191440352791947
**Ccl3**	5.41894553210761	2.32754971492484e-141	2.19720693088905e-137
**Gm19244**	5.36588719363591	0.00111060366661909	0.00369483651555392
**Gm29747**	5.33494316236061	0.00454212777049383	0.0129775079156967
**Il10**	5.32370545948603	4.53040512543225e-18	1.4091276568066e-16
**Hamp**	5.28696883626097	3.5252515920275e-11	4.64457432362032e-10
**Gdf1**	5.25790648174608	0.00127610997137646	0.00418280490617839
**Serpina3f**	5.24221133875589	3.25526467727613e-11	4.31294014785778e-10
**Gm5796**	5.20759777316914	0.000273494369224298	0.00104972020552038
**Gm12889**	5.1922070850188	8.097890702734e-05	0.000349938604869805
**Lbx1**	5.12080835736297	0.00282377817727402	0.00850557306747503
**Gm11662**	5.11139729924088	0.000153379476983582	0.000621017483476308
**Gm49134**	5.09196740042266	0.000174167264423491	0.000696669057693963

Table S5: *P. aeruginosa* vs Control top 50 down-regulated genes.

	log2FoldChange	pvalue	padj
**Gm10401**	-18.8815288366725	9.22253566888177e-06	4.86101265852842e-05
**Cldn2**	-6.49398396316403	3.26921629735514e-05	0.000153310491043381
**Gm10297**	-6.37156283941567	1.26192980037247e-06	7.91799090429783e-06
**Ccn5**	-5.58893945820872	6.6596619707845e-31	6.54866760460476e-29
**Ccdc121**	-5.28811272165018	5.32693853591002e-05	0.000239116974698006
**Cyp4a12a**	-5.2782179929384l	0.000336582248674833	0.00126763871034926
**Gm49510**	-5.24083036779679	4.07411678869835e-05	0.000187288349088446
**Gm7847**	-5.18674218703858	0.00178273971044423	0.0056463891516838
**Nlrp1b**	-5.13313423781666	0.0140750988798481	0.0349793164211573
**Galnt5**	-5.06770904605906	0.00450496616439325	0.0128967037427967
**Gm4202**	-5.04826704095145	0.00809887767844468	0.0215058805301035
**Alox15**	-4.88084806230365	0.0145083221772617	0.0358953115852053
**Cav3**	-4.72037957733482	0.000683850248803288	0.00240474812765991
**Syt15**	-4.62933733808203	3.45575492227435e-26	2.26543933793541e-24
**Pianp**	-4.62097399151396	0.00123789435854265	0.00407523025096518
**Gm9747**	-4.61223164589002	0.000977671653099535	0.0033020466566224
**Mansc4**	-4.56323273970713	0.00822861355455993	0.0217982634923659
**Chst5**	-4.54752767257629	6.54149902049228e-06	3.55712849962253e-05
**Rbbp8nl**	-4.3631530658707	0.00219563449157492	0.00681466039798364
**Calcoco2**	-4.28255963627702	0.00304937114638975	0.00912395043483971
**Htr2c**	-4.24241966781973	0.00611410168764386	0.0168566354939714
**Arl14**	-4.17384394399966	0.0113806494518689	0.029075326339822
**Sbk2**	-4.07337398094791	0.0116570861149519	0.0297132153166317
**Serpinb12**	-4.06104364757977	0.0187888661844655	0.0449532312027988
**Vmn2r44**	-3.97809551741671	0.014081030526959	0.0349894520069735
**Cd200r1**	-3.96038545819086	4.39617433354292e-24	2.46290122899971e-22
**Higd1b**	-3.9597666935667	1.10407342166932e-06	7.00433676112797e-06
**Gm4847**	-3.95860951998799	0.0206609617798725	0.048882074987969
**Pnmal2**	-3.93417276602414	1.49696509228847e-07	1.1118293053661e-06
**Gm46432**	-3.90940547371629	0.0174181367857399	0.0420529952064923
**Dmrta1**	-3.90144754891106	0.0176007281570066	0.0424721047551489
**Syt10**	-3.89838708235274	0.00489474265480414	0.0138570612269759
**Sat2**	-3.89284701701457	8.78887517078921e-06	4.64410756295831e-05
**Aplnr**	-3.88031655189699	2.82055179836755e-34	3.41359089443457e-32
**Fam171a2**	-3.87917712171314	1.73931256668298e-41	3.38538363494583e-39
**Gm47628**	-3.86786468647866	0.00993418279040218	0.0257456926675076
**Gm18095**	-3.85731376564362	0.0149378677604341	0.0367893221128353
**Ucp3**	-3.84670120372068	5.17390313024451e-08	4.20686008178365e-07
**Capn3**	-3.84582528970927	0.0163040445947797	0.0397085090234057
**Fam13c**	-3.82554227346075	2.75588711487645e-32	2.97320849879242e-30
**Vstm4**	-3.79312162223455	1.30336257358568e-39	2.25756746690805e-37
**Lrrc26**	-3.77338497803187	3.15477664870484e-05	0.000148608241336196
**Gapt**	-3.76502995790515	0.0013325958873734	0.00435283916152419
**Fam162b**	-3.73937873533203	0.00249908389360878	0.00763103734616428
**Kcnk3**	-3.70638870656352	7.96648482504419e-10	8.56045722804976e-09
**Cd300lb**	-3.70477859778041	4.40931536433233e-16	1.09825691396562e-14
**Thegl**	-3.65244623829845	0.00815611313283131	0.0216365625892729
**Tcrg-C2**	-3.63896719343264	0.0192803823367709	0.0459613154694739
**Lrat**	-3.6232724144124	8.07851567775344e-09	7.44374699833992e-08
**Wscd1**	-3.60170160819789	4.16908361517935e-18	1.30318375255937e-16

Table S6: *S. mitis vs* Control top 50 up-regulated genes.

	log2FoldChange	pvalue	padj
**Il22**	9.99668144147941	1.0179731246786e-09	5.09985206860347e-08
**Il12b**	7.22524986086376	4.582236772088e-16	8.06015448210279e-14
**Prkcg**	6.97574425155628	1.87781021933126e-06	4.17522662671814e-05
**Ifng**	6.70207685615832	8.42682419735348e-05	0.00109138722823718
**Olfr1039**	6.6791240092771	1.65044127912291e-05	0.000276195939638422
**Npcd**	6.57806952080447	0.000302365238004618	0.00305277046100199
**Il17a**	6.22516058223653	1.00099298574314e-07	3.1949032171975e-06
**B230303A05Rik**	5.32430439584196	0.000266201098262078	0.00276314601123156
**Ctla4**	5.28443543045021	0.000614918853048737	0.00550298494212241
**Csf2**	5.23766067429445	5.77078625117096e-63	1.14196646427859e-59
**Cxcl2**	5.1509166549735	2.53132253093284e-37	3.0825666913229e-34
**Il1b**	4.9411047847023	1.76732689878012e-174	2.79785521345881e-170
**Ccl4**	4.89400159478127	1.11170326776086e-55	1.95548604799135e-52
**Hamp**	4.89265847391955	9.08183009433438e-10	4.65289489396141e-08
**Kdm4dl**	4.88165358208999	0.00173435594317922	0.0127764490165055
**Il10**	4.8576733116022	2.90250094742808e-15	4.37614214273656e-13
**Il1a**	4.8252894807602	2.05738177963796e-82	5.42840182557476e-79
**Tnf**	4.69675960080689	7.96855140888222e-112	6.30750686770072e-108
**Il6**	4.66529043708641	3.15356504701218e-85	9.98481765184996e-82
**Ccl3**	4.48160480763072	3.97471816921358e-97	2.09745877789401e-93
**Csf3**	4.36756820412707	3.86839904112566e-33	2.78366478273002e-30
**Lif**	4.35925281768172	1.9474480760455e-79	4.40429292741091e-76
**Il12a**	4.31194772801463	4.2168167919587e-06	8.5366274467389e-05
**Cxcl1**	4.06475427156858	1.49795909764984e-51	2.37141904748947e-48
**Clec4e**	4.04238516469159	5.36864327224572e-35	4.2495495821461e-32
**Il1f6**	3.92251783688422	0.00466797374035672	0.0276670506490405
**Ccin**	3.87618474241021	4.96197089309802e-05	0.000705143278353992
**Il11**	3.79477411539678	1.46871155823705e-36	1.29173181546949e-33
**Fgf23**	3.54638235296698	1.60845782119787e-07	4.87806432325353e-06
**Gm11843**	3.52685853909233	6.44718910373525e-06	0.000122970422531606
**Cd70**	3.49487879585607	0.00975564447301435	0.0486124040454171
**Pou3f1**	3.48412497173064	1.43620109153419e-07	4.40379369337945e-06
**Cemip**	3.43523530361376	3.42986537139022e-09	1.56931210099649e-07
**Ccl20**	3.41998530685403	1.88778957943728e-05	0.000308416891971843
**Gm8752**	3.32861102833156	3.45610573359014e-09	1.57676109131025e-07
**A730049H05Rik**	3.31874011557085	2.9217903415584e-18	7.22732232768922e-16
**4930548H24Rik**	3.27052568702648	0.000109891399604521	0.00136446333108955
**Acod1**	3.17558630845271	7.00472363568804e-11	4.5634477315464e-09
**Serpinf2**	3.10415372724911	0.000697810419698092	0.00610670909576589
**Cxcl11**	3.10124667126266	3.609076781743e-12	3.1919158956298e-10
**Ptgs2**	3.09122909060085	3.43027101039033e-16	6.10164273769543e-14
**Cxcl3**	3.06880917453999	2.49206835888549e-38	3.28766118245968e-35
**Gm20708**	3.05761156709634	0.00689643499120615	0.0374430518325526
**Zfp819**	3.01562237043984	2.13572573827668e-05	0.000341716188437312
**Nlrp3**	3.00968810524825	1.56612601112103e-13	1.8926214413784e-11
**Slamf1**	2.80596106677752	8.74095482525593e-10	4.52138289023649e-08
**Tarm1**	2.75960711453636	6.83287614812416e-16	1.18869519012037e-13
**3425401B19Rik**	2.75606446719998	1.24478751164992e-05	0.000217989282045684
**Gli1**	2.73931097967432	3.59898935444947e-18	8.76547699542917e-16
**Hcar2**	2.73609588698318	1.67038077881446e-08	6.44970685595408e-07

Table S7: *S. mitis vs* Control top 50 down-regulated genes.

	log2FoldChange	pvalue	padj
**Lrat**	-3.96661151887988	2.83881238674999e-10	1.61079709299782e-08
**Ccn5**	-3.91488483062592	2.85655549591764e-17	5.79770898152207e-15
**Sprr2a2**	-3.45439671776039	0.00824951803086746	0.0430419988098407
**Higd1b**	-3.41644121022509	1.31777081335489e-05	0.000227747049631236
**Aspn**	-3.12988959164833	8.72320301540876e-08	2.83212851405143e-06
**Cd244a**	-3.10765404186874	5.85410939049655e-05	0.000807285764468213
**Htra3**	-2.82460094192981	4.95761164522097e-11	3.32559109980903e-09
**Gpr27**	-2.73331240219781	0.00273331978038129	0.0183274821868768
**Sstr2**	-2.47427454759749	6.92148170366006e-08	2.30682056527668e-06
**Tril**	-2.4438065759647	1.25073265479213e-09	6.14917660186775e-08
**Eln**	-2.44039659211252	6.26275159305576e-34	4.72122002236503e-31
**Tmem273**	-2.42895014215316	0.00010585371894139	0.00132367316316047
**Glod5**	-2.34129462066259	9.18759160327075e-06	0.000167954691306443
**Thbs2**	-2.34021483815627	5.24498606462701e-13	5.32265220443014e-11
**Kank4**	-2.33027158670107	7.12937402461012e-05	0.000946061359460208
**Olfml1**	-2.29984507234638	0.000186949294403116	0.00206964634943757
**Slco2b1**	-2.23798165065996	7.88800486914268e-13	7.70833364712332e-11
**Cmya5**	-2.22563107193905	1.16337298415191e-07	3.62546411655686e-06
**Ildr2**	-2.21374141994243	3.10158704224008e-25	1.25900575553084e-22
**Rgs4**	-2.19805574705384	3.15025043921211e-06	6.58806006646856e-05
**Igf1**	-2.18782136173475	4.89504953486301e-22	1.76121657241855e-19
**Adamts15**	-2.17829996307745	4.48369158729542e-06	8.98497740740175e-05
**Chst5**	-2.17689855128104	0.000869921198693421	0.00732148989713745
**Xntrpc**	-2.17245673337296	3.56970836570156e-05	0.000531128318960727
**Sfrp2**	-2.16980390663505	0.000420275438871558	0.00401531712297866
**Cd180**	-2.11992780637565	4.05720762683147e-10	2.23796703624979e-08
**Adamtsl3**	-2.11443664088227	1.90582965919296e-07	5.65003545593327e-06
**Serpina3n**	-2.07089677074857	1.04796387601682e-18	2.67585743890682e-16
**Syt15**	-2.03301805061583	1.51618941570255e-11	1.15398051153784e-09
**C1qtnf2**	-2.02430960088116	1.93347353323313e-06	4.28095377686905e-05
**Rarres1**	-2.00730276712228	5.47877422267501e-11	3.64430566046925e-09
**Zfp784**	-2.00554162355129	0.00871155980801613	0.0446319428222341
**Galnt15**	-2.00269021447912	1.10447304719405e-36	1.1439936213618e-33
**Fam13c**	-2.00195531566993	9.47496232489917e-13	9.14622735155358e-11
**Kcnk3**	-1.99933947256829	0.000431273339249544	0.00409567380543463
**Krt79**	-1.97242576467205	1.33052540644716e-07	4.11397416200489e-06
**Cxcl12**	-1.96255199574218	6.23371671890987e-11	4.07793261888687e-09
**Apln**	-1.96152360655464	6.32133134989045e-06	0.000120861107004971
**Rab3il1**	-1.94315372772449	2.92644034527281e-07	8.11356867005495e-06
**Kirrel3**	-1.93263697357184	0.00116509992031236	0.00928268587743579
**Ccdc85a**	-1.92244173596888	2.70009944023802e-10	1.55066984194034e-08
**Lalba**	-1.9219583797168	2.81591218872314e-06	5.96769824092049e-05
**Sirpb1c**	-1.91276822449401	0.000621317395075046	0.00554770201998481
**Svep1**	-1.90968811729934	8.49534333635372e-08	2.76727943123078e-06
**Card11**	-1.89518247007166	2.78598347193071e-10	1.58650735050845e-08
**F2rl3**	-1.89427282468333	4.67284624618e-09	2.0606080480021e-07
**Pcdh18**	-1.89091886645232	3.43958857514276e-05	0.000513215143572904
**Abca9**	-1.88457071082673	0.00048041618753747	0.00448435652411892
**Vstm4**	-1.88379647423305	7.23137395386812e-17	1.41333186498378e-14
**Scml2**	-1.86669107066618	0.00960133777455254	0.0481138129960203

Table S8: ***S. mitis*** **vs** ***P. aeruginosa*** **top 50 up-regulated genes.**

	log2FoldChange	pvalue	padj
**Gm10401**	19.8352988080259	3.11871929414909e-06	2.67857405472264e-05
**Gm10297**	6.87683311482651	1.66872511612049e-07	1.90607219974808e-06
**Gm49510**	6.37152676458894	4.85149288077773e-07	5.00822038653044e-06
**Ccdc121**	5.43257851568077	3.37705090149534e-05	0.000225837095543062
**Cyp4a12a**	5.17988405190126	0.000462076890467817	0.00223538833945628
**Calcoco2**	5.02332379078133	0.000460704102069196	0.00222935532065408
**Trpc2**	4.96490689612507	0.0121221548537168	0.0357001687696227
**Sbk2**	4.80978895229431	0.00269933764253942	0.0101323639349633
**Pianp**	4.76874527963311	0.000888795056071766	0.00390361077747221
**Gm10849**	4.67666790831937	0.0071819014441959	0.0230577520117332
**Gm15697**	4.51786891134309	0.00937558881873764	0.0287996589888969
**Cabcoco1**	4.47960709465844	0.00589762788670801	0.0195102463291861
**Gm18283**	4.34322005621316	0.0152042493220337	0.043323804904606
**Cldn6**	4.14747285830901	0.000352554918253481	0.00176461043258931
**Gpr62**	4.10920801354043	0.00361866709437504	0.0129729251907704
**Rbbp8nl**	4.07693520559656	0.00451766641908037	0.015651653436685
**St8sia3**	4.04415967088334	0.00305578433657837	0.0112483053568322
**Fbxl13**	3.96879890087694	0.00748705857391673	0.0238775370991572
**Thegl**	3.72998274649254	0.0071625438021742	0.0230039323309597
**Kcnab3**	3.71817326212668	8.07743418099131e-09	1.24351818749897e-07
**Cldn8**	3.71016618773602	0.0111868519883901	0.0333331350830139
**Sat2**	3.68086748673448	2.97539842665475e-05	0.000201869514524568
**Tigd4**	3.60564814757661	0.00972273497232578	0.0296888265726924
**Syt10**	3.59647988032001	0.0102859047520499	0.0311138531089213
**Ccdc160**	3.59457237399591	1.11252504233646e-06	1.05511399068647e-05
**Gm49383**	3.54415725090895	1.25641959829758e-06	1.18142610287432e-05
**Nhlrc4**	3.51380253677588	4.80925155607161e-08	6.17164083552316e-07
**1500015O10Rik**	3.48596654658042	0.014909140144734	0.0426267290773589
**Stpg1**	3.47835871740297	0.00384297124154096	0.0136555990720755
**Pif1**	3.4530828855647	8.52036521008872e-05	0.000508783424245534
**Gm48515**	3.41938162984523	0.00391188150839335	0.0138560452469255
**Fam171a2**	3.38986599480917	1.89839338020407e-31	6.8710217546774e-29
**Gm29793**	3.38568639891546	0.00752402200666369	0.023978172558523
**Ankrd63**	3.34530373169706	7.63822837710482e-07	7.50077410121561e-06
**Sla2**	3.28524078961477	0.00228374046098313	0.00876859430083043
**Slc40a1**	3.25263543005238	8.73685470628854e-46	1.93685147770034e-42
**Tlr8**	3.23189452324867	1.47756012244854e-19	1.27826969617683e-17
**Prrt1**	3.21264332977906	1.32852136101375e-26	2.58915674039327e-24
**Frmpd2**	3.20155562192182	1.94852145094244e-05	0.000137677402121371
**Clec4a3**	3.19602140154224	4.61994320054303e-10	9.01371756453582e-09
**Sh2d1b2**	3.14097350941079	0.0152443776988615	0.0434241950673481
**Prss30**	3.10769080733856	0.003746886163194	0.0133631553082808
**Gm18009**	3.08355095682484	0.0080682207580765	0.0253390995474565
**Cd200r1**	3.02402784968762	3.25154790208626e-14	1.30172013642212e-12
**Tctex1d4**	3.01388934893705	0.000206302412154412	0.0011048450661544
**Gm13288**	3.01010620512287	0.00596537196209747	0.0196939448525314
**Slc16a5**	2.96555523846774	9.2335655963317e-07	8.92410277116854e-06
**Capn12**	2.9601530131972	0.00074200559990942	0.00335615131711134
**Cysrt1**	2.91023381582525	1.55670186654509e-06	1.44017254059349e-05
**Klhdc7a**	2.90648939330121	5.43176014021545e-19	4.35892606727244e-17

Table S9: ***S. mitis*** **vs** ***P. aeruginosa*** **top 50 down-regulated genes.**

	log2FoldChange	pvalue	padj
**Gm8024**	-9.24604322405623	9.28613636703366e-06	7.0651921265269e-05
**Gm6482**	-8.83470401450131	1.78778295698007e-11	4.54245426103747e-10
**Gm3141**	-7.77805111672937	1.27188408290094e-07	1.4859594341402e-06
**Igkv15-103**	-6.52142120769322	0.0017972680773655	0.0071467599444119
**Lep**	-5.91393693994332	0.000330178265073357	0.0016640271472225
**Rhox4b**	-5.84871694398225	0.00535203656340743	0.0179769637219755
**Sprr2d**	-5.64378390794899	5.12828843255043e-05	0.000326220212881212
**Serpina3h**	-5.38289750771718	9.29459284835061e-05	0.00054836860999833
**Gm47036**	-5.13400350403633	0.0178163409858735	0.0493399137077557
**Cxcr1**	-5.01048054334826	0.000445019996648343	0.00216290206647256
**Ugt1a9**	-4.89918212862831	0.00203574972430798	0.00796648686991729
**Lgsn**	-4.89558567498541	0.000417499754875166	0.00204766541833824
**Frem2**	-4.85749346851223	0.00174459001293707	0.00696383161814966
**Gm10155**	-4.75169177396807	0.00700139398769198	0.0226009687607785
**Tph1**	-4.7148041234223	0.000755956206293117	0.00340812580881337
**Alms1-ps2**	-4.69799950158542	2.20191064839937e-05	0.000154012277780238
**Gm43058**	-4.653065641366	0.000778674217512932	0.00349349538264403
**Gm8094**	-4.64363953022242	0.00161132687492591	0.00649327019468551
**Slc32a1**	-4.44617995250715	0.010604767414448	0.0318782249031456
**Tmem132e**	-4.24859252811628	7.12513192219173e-08	8.83665836643848e-07
**Cd177**	-4.15113308116961	1.95632800376471e-05	0.000138118937686175
**Il13**	-4.05632791525347	2.298685189648e-05	0.000159746010338586
**Tbx15**	-4.05256687706085	0.00460994256676522	0.0158999088723417
**Pglyrp2**	-3.92668649056925	1.76540950598002e-17	1.15108594075572e-15
**Gm12889**	-3.91585131695621	0.000424231921341633	0.00207437362144854
**Unc93a**	-3.88341417282021	0.0104513279956291	0.0314960581142704
**Dbx2**	-3.86971639325264	0.00364003673508984	0.0130310963861159
**Depp1**	-3.8148745912203	5.0069272763094e-06	4.08829904444509e-05
**Gm8281**	-3.76430033709364	0.00026923135543377	0.00139778047090688
**Cited1**	-3.76267521455097	9.62044648017313e-05	0.000565524091235898
**Gm18301**	-3.75809799189445	0.00176400768740287	0.00703184453497187
**Fam180a**	-3.74941268053223	0.00989270685334769	0.0300990145898304
**Tmem74b**	-3.68258643972699	0.0120958203239505	0.0356403677430241
**Cnr1**	-3.62721457130931	1.05825566224434e-19	9.4788707928805e-18
**Fam19a2**	-3.45881267813742	0.0101750325074809	0.0308363297197838
**Duox2**	-3.44443122652896	9.92035560199062e-06	7.49946745956111e-05
**Trim66**	-3.41704484082984	3.51267060507175e-08	4.70972617954402e-07
**Olfr329-ps**	-3.36137806128127	0.00768390548526936	0.0243825485384241
**Slc5a8**	-3.33858657708622	0.000816395608448487	0.00363240745505116
**Ccl20**	-3.29141020364826	2.02455887338496e-05	0.000142425829510045
**Sele**	-3.28507484917569	4.72373921464344e-49	1.39625858286169e-45
**Nat8f3**	-3.27807645756005	3.02780510236361e-21	3.37724047109551e-19
**Hist1h3d**	-3.2127181109906	2.79316206673874e-05	0.000191040220800662
**Gm3739**	-3.19729122341784	0.000614632928963361	0.00286027682895964
**Serpina3f**	-3.14943337817975	6.32890003406803e-05	0.000392183934675739
**Pthlh**	-3.09296945644182	0.00279893999871928	0.0104437620192061
**Stc2**	-3.08676678329759	6.8598757058303e-11	1.55376622787868e-09
**Gm8288**	-3.06705099143864	8.56003022338142e-05	0.000510807994655685
**Tslp**	-2.99627623308518	1.18770449464056e-16	6.8612179845115e-15
**Gm3696**	-2.98993340084993	0.000163875519468915	0.000900350786177575

Table S10: Co_Infection vs *P. aeruginosa* top 50 up-regulated genes.

	log2FoldChange	pvalue	padj
**Gm10401**	21.1890798347379	6.1715777308667e-07	1.09634708469529e-05
**Gm49510**	7.20070670941502	1.11071222222517e-08	3.19946826235416e-07
**Gm10297**	6.28948633787759	1.80186133029562e-06	2.79406384974438e-05
**Olfr1425**	6.24790541861278	0.000154801263103327	0.00129654848935406
**Pianp**	5.74947933365525	4.95890884321803e-05	0.000492389081548017
**Alox15**	5.41375450208552	0.00669619028484584	0.0277323569642019
**Muc5b**	5.15739991106209	0.00172676463177484	0.00946810270006876
**Olfr876**	4.99478008794211	0.0022166284807807	0.0115546024986219
**AC161519.2**	4.84318830566471	0.00644544150814792	0.0269241604992926
**Gm18997**	4.8348586067205	0.00201273286823463	0.0107134630494685
**Gm47628**	4.75937570879691	0.00142475104687578	0.00807358926562941
**Olfr1543-ps1**	4.61259957275726	0.00287186644949612	0.0142305650215417
**Calcoco2**	4.59772503967728	0.00150680179587744	0.00847486623633847
**Ccdc121**	4.19259468947876	0.00163995793825316	0.00908823717818853
**Gm29257**	4.15950003849399	0.000197620132988594	0.00160652290374657
**Lrrtm4**	4.07133085467618	0.00085818356830741	0.00531449516287348
**Gpr62**	4.01048736766741	0.00465954636844823	0.0208949171203495
**Gm9696**	3.9742682227883	0.000377872176352514	0.00274743871605649
**4930578G10Rik**	3.87776845322361	0.000976665325811853	0.00590124948502195
**Gm8454**	3.7048237457673	0.00229495511896461	0.0119067840318514
**Gm43941**	3.6802123237402	0.00029583669807863	0.00225058344544733
**Olfr933**	3.63517721826783	0.00753002292040043	0.0303690180591356
**Gm48515**	3.58751589461991	0.00244190962637594	0.0125088498057516
**Fam162b**	3.54258912733721	0.00434598661319671	0.0197125778803061
**Olfr102**	3.51499158556776	0.000439851912593058	0.00311614984361401
**Hoxb8**	3.49980748747981	1.56463802240911e-11	8.86023005727369e-10
**Sat2**	3.4875099814373	8.27935535637216e-05	0.000758956155099043
**P2ry4**	3.4330119356479	0.00494636398221024	0.0219028746177828
**Gm15217**	3.3523585781318	0.00499180177148989	0.022051138422668
**Obox3**	3.34699016865037	2.60472610401314e-06	3.83474849314872e-05
**Kcnk3**	3.28888936654426	5.40952210929584e-08	1.29703454967394e-06
**Gm3867**	3.19014671892549	0.0121329861800103	0.0439830689750339
**Baiap2l2**	3.14629224631155	0.00666732078968321	0.027656046635606
**Gm3755**	3.14143916192378	7.90164195990678e-05	0.000729977969926355
**Pou1f1**	3.13718879684844	0.0054061490625785	0.023420058811045
**Gm18009**	3.11348833176236	0.00755612306942292	0.0304594737531256
**Il22ra1**	3.10256522136498	0.0023329415032243	0.0120673607498506
**Lypd2**	3.07829174342825	0.000912831290918774	0.00559294563475786
**Fam163a**	3.04998695132056	0.00919105121476177	0.0354069936743272
**Vmn2r104**	3.03075068004047	0.00413643584398602	0.0189905211741605
**AC116766.1**	3.0216751946022	2.05314701601554e-05	0.000232531162387235
**Olfr825**	3.00613193062201	0.0111581272342658	0.0411870182582732
**Gm11549**	2.99737670635679	0.0030535578569073	0.014923308383448
**Gm14667**	2.99089888422865	4.87660767054259e-05	0.000488013718152247
**Tulp1**	2.9639109939213	0.000688321183153343	0.00444555277184907
**Hoxb1**	2.96023154076421	0.000954210038598339	0.00580786975804242
**Ly6g6d**	2.94982300606174	4.73519363482374e-07	8.64316092288179e-06
**Thrsp**	2.94079706173902	0.00468246505221134	0.0209804439253666
**Gm20708**	2.90051564423174	0.00605554135523725	0.0256834207991044
**Cntn3**	2.89736069583009	0.00032152524850087	0.0024128208607537

Table S11: Co_Infection vs *P. aeruginosa* top 50 down-regulated genes.

	log2FoldChange	pvalue	padj
**Gm42417**	-5.62001288984596	0.00743161312889644	0.0300232103809714
**Sirpb1a**	-4.60564706754273	0.00107783055416263	0.00640980808410983
**Ugt1a9**	-4.36961818184354	0.0044153442515405	0.0199834674908783
**Gm6482**	-3.787270656297	2.58687821565242e-08	6.76995005585252e-07
**Fam180a**	-3.65726047135965	0.0121309367666081	0.0439830689750339
**Gm14412**	-3.4925662062958	0.000592108888462598	0.0039328954642644
**Gm49393**	-3.33281550295654	0.00580507654833053	0.0248369855827489
**Fam189a1**	-3.2869753168242	1.08397377388525e-05	0.000134218603405255
**Igkv1-117**	-3.08131536439125	0.00704427083597634	0.0288517752926486
**Alms1-ps2**	-3.0661581394557	0.00167758641930217	0.00924120646383187
**Mmp1b**	-3.01466081696545	0.00192925522377187	0.0103344755742316
**Cnr1**	-2.85661494639036	1.46216452094413e-13	1.19508540549286e-11
**Nat8f3**	-2.81088422673094	3.65480005125607e-17	5.41432521878934e-15
**Slc6a12**	-2.66628062967039	1.62350427707408e-10	7.18324879072352e-09
**Trim66**	-2.52317773553632	4.88325510048201e-06	6.68506341808561e-05
**Slc6a19**	-2.43642812188425	4.03638555555089e-08	9.99577748323886e-07
**Chgb**	-2.36136718017517	0.00217405122092907	0.0113540629076661
**Actn3**	-2.35596462127843	0.00468043732614127	0.0209772598906904
**Gm8281**	-2.3371573804433	0.0130342496595773	0.0463589003969362
**Hist1h3d**	-2.32262946624283	0.000645942821779738	0.00421948161376749
**Rps7-ps2**	-2.31683063093943	5.30356042126722e-05	0.000520074908449561
**Tlr7**	-2.18456395524982	1.27408833735684e-09	4.76118776878936e-08
**Plcxd2**	-2.17396461881146	2.44728904274664e-43	1.35351399324174e-39
**Slamf8**	-2.16790932523167	1.32159812352551e-14	1.2898797685609e-12
**Stc2**	-2.1185408589092	7.02217334305422e-06	9.18863565520155e-05
**Il13ra2**	-2.10610888581696	4.0361595839833e-09	1.31399119813391e-07
**Gm9042**	-2.09031461678602	0.014169815118966	0.0494834636655955
**Gpr84**	-2.07756855993534	1.78702017894152e-06	2.77364254527575e-05
**Apobr**	-2.07138045110051	1.33118558170375e-10	6.03470797039032e-09
**Adam4**	-2.06407708679617	0.00858560127966463	0.0335931957292851
**Gm20056**	-2.05316963271651	0.00920328532294632	0.035429445493811
**Ankrd22**	-2.04415126870789	0.00507958153796406	0.0223068869167102
**Bcat1**	-2.04092823647141	1.63245583742877e-13	1.30848827316996e-11
**Pglyrp2**	-2.03860910380172	6.14729236308854e-06	8.17567861086082e-05
**Ephb1**	-2.00509188846146	0.0034174301498286	0.016350057971729
**Gm8822**	-2.00278212925597	0.0114978076296931	0.0422061115468733
**Slc25a18**	-1.98783127086461	0.00395565155837868	0.0182972318529744
**Scn11a**	-1.98440305294084	0.0106608641366318	0.0398659133998187
**Gm8941**	-1.96811999028636	0.0104345443998081	0.0391874062203747
**Nrk**	-1.962214641368	0.00238786565667232	0.0122927294370174
**Pkd1l1**	-1.95386777280302	0.00928036976588965	0.0356765280712792
**Bend6**	-1.95104655497121	0.000252600494093981	0.00197416269336191
**Gm15009**	-1.94806886455059	0.000605303923936991	0.00400287074769333
**Galr2**	-1.94105596674227	0.000476347049277194	0.00331663879211381
**Slc7a1**	-1.91828424193005	8.60796563116399e-25	5.28975428712122e-22
**Mthfd2**	-1.88622286058261	1.25339929315974e-17	2.05904961109964e-15
**Igsf6**	-1.88176029510809	0.000565900161985041	0.00380160988753667
**Madcam1**	-1.86212800377449	0.000187539701641402	0.00153359227680342
**Kansl1l**	-1.85436366975263	8.78481156250134e-16	1.07968587737054e-13
**Garem2**	-1.85294824871954	4.75305929750502e-07	8.66623734771464e-06

Table S12: Co_Infection vs *S. mitis* top 50 up-regulated genes.

	log2FoldChange	pvalue	padj
**Gm8024**	-7.19086755791243	0.000582131053074662	0.00675513708045337
**Sprr2d**	-6.28606534364153	6.07571975173963e-06	0.000162530516329127
**AC161519.2**	-5.95211947024691	0.00133716643500571	0.0131775590545345
**Gm47036**	-5.9446603368256	0.00572084897879176	0.0392968994894886
**Gm3141**	-5.69122780033145	0.00013610181417975	0.00213379739813145
**Olfr876**	-5.22971262245137	0.00122994862281373	0.0123689188116833
**Olfr1425**	-5.06801536035075	0.000379523148558935	0.00483303201986558
**Gm6482**	-5.04743336035739	0.000198485099140181	0.00286777356698883
**Muc5b**	-4.50924505637652	0.00606370532167409	0.0409406056005353
**Gm3867**	-4.14014228570082	0.0024114852983938	0.0206511295151938
**Olfr102**	-4.08817607569109	7.23649453528889e-05	0.00126370921218827
**Gm18301**	-4.05225997285113	0.000663618210716077	0.0075165228760156
**Lrrtm4**	-3.96853252213108	0.000561894297230865	0.0065815523500069
**Nkx6-1**	-3.93403288408663	3.65816284217666e-05	0.00070935978270964
**Cxcr1**	-3.82501440751835	0.00790451533551212	0.049987780721363
**Depp1**	-3.76471212553056	6.65196175154312e-06	0.000175626957580237
**Il23a**	-3.47399656879125	5.32407444272505e-14	1.13563908935547e-11
**Ccl20**	-3.4662472477893	7.15180020204683e-06	0.000186395230024729
**Gm4297**	-3.46394962555	0.00771901512715127	0.0490759792075394
**Duox2**	-3.42531109303143	1.09517579900054e-05	0.000261856856601738
**Gm8454**	-3.34852309166316	0.00362634829272706	0.0281680556652604
**Gm12889**	-3.3470577478301	0.00272641240266443	0.0226076068847023
**Hoxb1**	-3.24378465953908	0.000289894019123164	0.00388065395871645
**Edn2**	-3.24184721353493	0.00224891371647409	0.019590080740334
**Obox3**	-3.19271012628584	4.92627247376182e-06	0.000137216156481362
**Gm43941**	-3.17583936629095	0.000833420086622268	0.0090371725914606
**Duoxa2**	-3.11887465695351	0.00244139294997458	0.0208021119102532
**Gm9696**	-3.11817795682428	0.00147331697691417	0.0141660388569132
**Il13**	-3.02113813136482	0.00192897238413254	0.0173628935697793
**Cntn3**	-3.01136808902619	0.000152504665316079	0.00234781873640926
**Grip2**	-2.97967226918598	0.00374082600891184	0.0289187078829136
**Gm14735**	-2.94973226360186	0.00264365890140745	0.0221137019869536
**Cited1**	-2.92988660162311	0.0025323838882494	0.021361770696657
**Adra2b**	-2.92888418751246	0.00238183427277709	0.0204512263749944
**AC116766.1**	-2.92108828823995	3.43532021567465e-05	0.000675849223498807
**Sele**	-2.90625394112157	9.5051341809658e-39	7.70438651038183e-35
**Gm18716**	-2.90023801787908	0.000628745265193159	0.00716778445432229
**Csf3**	-2.87628019307875	9.86652903153058e-16	3.07589042557966e-13
**Gm13889**	-2.87014063305351	7.00098441027109e-19	3.43918055378499e-16
**Sp8**	-2.85732617994959	0.00769912305381659	0.0490221853202752
**Rnd1**	-2.7729888159655	4.6940105286466e-37	2.536486822663e-33
**Olfr1250**	-2.75471568663498	0.0014757724058374	0.0141716622339293
**Tslp**	-2.72873374623126	4.55322561615295e-14	9.97464060316966e-12
**Olfr1254**	-2.72839687238863	0.000120248049761264	0.00191864284909434
**Gm3739**	-2.71382640632426	0.00385685397209745	0.029603910862534
**Olfr1160**	-2.67411650153816	0.000994131407443012	0.0104377359106598
**P2rx1**	-2.6624103379609	0.00614672544655579	0.0413634562947763
**Tmem132e**	-2.62292809763687	0.000992775266450905	0.0104377359106598
**Gm2564**	-2.61237256405827	1.91125485491754e-14	4.49034093522729e-12
**Gm2961**	-2.60146896891511	0.0054617503338568	0.0380165026458362

Table S13 Co_Infection vs *S. mitis* top 50 down-regulated genes.

	log2FoldChange	pvalue	padj
**Tlr8**	3.7923051110679	1.96942556829436e-22	1.59631789438099e-19
**Ear6**	3.01515903684952	0.000175996458330637	0.00262231487683635
**D330045A20Rik**	2.93572085655222	0.000759707235245228	0.00835909535977781
**Gm13288**	2.89191155414066	0.00616345827406363	0.0414244701827718
**Clec7a**	2.80955741687338	1.78853519643482e-33	7.24848601735122e-30
**Klra2**	2.74901057788716	5.27985997977875e-07	2.10299287794087e-05
**Cd200r1**	2.69620775605273	6.32817003335951e-14	1.31520467193322e-11
**B430306N03Rik**	2.6174548671745	2.3911132587461e-09	1.83707758471721e-07
**Zfp300**	2.61201323060577	0.00407707228383035	0.0308714318727378
**Pik3cg**	2.61188787158024	1.33779488839863e-29	3.09814184797574e-26
**Ear2**	2.61105340475918	1.25808636064125e-09	1.02486623077162e-07
**Fpr1**	2.60542503290577	8.28657273797401e-31	2.68667261310593e-27
**Tfec**	2.57488359099493	1.10651157183917e-18	4.98268308085689e-16
**Dio2**	2.53930849184064	8.74248227718447e-17	3.37439000465327e-14
**Abcd2**	2.50864631872662	2.07347094131421e-21	1.60062082998308e-18
**Cd200r4**	2.48630299273812	1.88110246132058e-11	2.43956416003744e-09
**Ccr1**	2.45151753806176	2.69077816025769e-06	8.24578539809782e-05
**Ccl6**	2.41238834776474	2.02018327632493e-27	2.72909925770862e-24
**Zfp108**	2.3983412829398	0.000322245747061983	0.00424019951755016
**Fam198a**	2.39405368966943	0.000951535153633349	0.0101282576333225
**Treml4**	2.37463167585583	4.98200200461704e-06	0.000138530419377096
**Scimp**	2.32364894348203	2.65747296859674e-12	4.18255284407008e-10
**Cd300lb**	2.31660393820902	2.93114626293281e-07	1.25903995668272e-05
**Gm49383**	2.2899292182963	0.000466105700647689	0.00567696432246408
**Alox5**	2.26540454873655	3.32218282592706e-08	1.96554400697458e-06
**Tnfaip8l2**	2.26505167825468	2.71082038470903e-10	2.58843927453579e-08
**Arl11**	2.2065835454338	2.81976349061658e-10	2.67316876879446e-08
**Syt15**	2.19934755698416	6.29910962035032e-08	3.43821097829963e-06
**Btk**	2.18299176374556	7.95470019605177e-13	1.37184728593825e-10
**Lipf**	2.15594582589831	0.0043575884905278	0.0323892100045604
**Lilra5**	2.12673341900353	2.36345301200383e-09	1.82447317988543e-07
**Fcgr1**	2.12518322029855	4.13344643031931e-12	6.14745872311068e-10
**Atp6v0d2**	2.08502052312783	7.59174856387066e-16	2.5639549160189e-13
**Gcnt3**	2.07352847171418	0.000112163828632663	0.0018182878259641
**Slc6a12**	2.04576377849669	1.06289698286456e-06	3.81208473212774e-05
**Creg2**	2.03681334296193	0.00104465200339766	0.0108626386318663
**P2ry13**	2.03368353066736	0.00598361076509774	0.0405859054866107
**Thbd**	2.02223116601951	1.8498952191373e-26	2.30681933826421e-23
**Srl**	2.01254364857783	4.62780188057531e-06	0.00012934706256208
**Mcoln3**	2.00288860140102	4.99943317281417e-08	2.78710782448407e-06
**March1**	1.98626228265442	3.24753718117496e-14	7.31192017278157e-12
**Cd300ld**	1.96848116377579	6.0528496098251e-06	0.000162186355413016
**Gm14048**	1.95886579409938	0.0022273517771076	0.019433584315765
**Fcgr3**	1.95833594334697	3.85986745582152e-08	2.23472540451153e-06
**Myo1f**	1.95340191006777	3.75689001055916e-25	4.0601962640783e-22
**Zkscan4**	1.94674737303137	0.000274093339618675	0.00369661158781892
**Cd33**	1.93128875241588	4.29861620457223e-11	5.20036323077018e-09
**Tlr13**	1.91719013440769	1.52353522701648e-06	5.12407252389299e-05
**Nfam1**	1.8936599950334	1.13852698489737e-09	9.33729779513466e-08
**Clec5a**	1.88700718720813	8.03313967341877e-09	5.54149903173581e-07

Table S14. Genome sequence information of *Streptococcus* type strains.

Organism_name	Strain_name	Assembly_accession	Bioproject	Biosample
Streptococcus salivarius	strain=NCTC 8618	GCF_000785515.1	PRJNA224116	SAMN03174835
Streptococcus sanguinis	strain=NCTC7863	GCF_900475505.1	PRJNA224116	SAMEA3672886
Streptococcus thermophilus	strain=ATCC 19258	GCF_010120595.1	PRJNA224116	SAMN11175069
Streptococcus mutans	strain=NBRC 13955	GCF_006739205.1	PRJNA224116	SAMD00169830
Streptococcus pneumoniae	strain=NCTC7465	GCF_001457635.1	PRJNA224116	SAMEA2479568
Streptococcus pyogenes	strain=NCTC8198	GCF_002055535.1	PRJNA224116	SAMEA2479569
Streptococcus intermedius	strain=NCTC11324	GCF_900475975.1	PRJNA224116	SAMEA4012327
Streptococcus uberis	strain=NCTC3858	GCF_900475595.1	PRJNA224116	SAMEA3871780
Streptococcus lutetiensis	strain=NCTC13774	GCF_900475675.1	PRJNA224116	SAMEA3905384
Streptococcus gallolyticus	strain=NCTC13773	GCF_900475715.1	PRJNA224116	SAMEA3881065
Streptococcus oralis	strain=NCTC 11427	GCF_900637025.1	PRJNA224116	SAMEA3936797
Streptococcus parasanguinis	strain=ATCC 15912	GCF_000164675.2	PRJNA224116	SAMN00113608
Streptococcus parauberis	strain=NCFD 2020	GCF_000187935.1	PRJNA224116	SAMN02436555
Streptococcus equi	strain=ATCC 33398	GCF_900156215.1	PRJNA224116	SAMN05421817
Streptococcus infantarius subsp. infantarius	strain=ATCC BAA-102	GCF_000154985.1	PRJNA224116	SAMN00000017
Streptococcus equinus	strain=ATCC 9812	GCF_000187265.1	PRJNA224116	SAMN00217012
Streptococcus agalactiae	strain=ATCC 13813	GCF_000186445.1	PRJNA224116	SAMN00217013
Streptococcus pseudopneumoniae	strain=CCUG 49455	GCF_002087075.1	PRJNA224116	SAMN06459226
Streptococcus dysgalactiae	strain=NCTC6403	GCF_901543725.1	PRJNA224116	SAMEA3649039
Streptococcus pasteurianus	strain=NCTC13784	GCF_900478025.1	PRJNA224116	SAMEA4030747
Streptococcus gordonii	strain=Challis substr. CH1	GCF_000017005.1	PRJNA224116	SAMN02603977
Streptococcus macedonicus	strain=ACA-DC 198	GCF_000283635.1	PRJNA224116	SAMEA2272145
Streptococcus iniae	strain=YSFST01-82	GCF_000831485.1	PRJNA224116	SAMN03286870
Streptococcus suis	strain=S735	GCF_000294495.1	PRJNA224116	SAMN02604110
Streptococcus mitis	strain=NCTC12261	GCF_000148585.2	PRJNA173	SAMN02435817

Table S15: Comparison of *S. mitis* genomes.

Strain	Total length	GC (%)	percent_completion	Num. genes
**SM1**	2022463	39.76	100	1841
**SM2**	1999481	39.96	100	1803
**SM3**	2827339	40.71	100	2092
**SM4**	1874967	40.28	99	1810
**SM5**	3517890	39.58	96	2026
**SM6**	2012296	39.96	100	1844
***S. mitis*_NCTC12261**	1868883	40.45	100	1690
